# Impacts of illegal trade on socio-emotional and behavioural skills in macaques

**DOI:** 10.12688/f1000research.144232.2

**Published:** 2024-11-29

**Authors:** Amapola Rey, Maria Padrell, Miquel Llorente

**Affiliations:** 1 Fundació UdG: Innovació i Formació, Universitat de Girona, Girona, Catalonia, 17003, Spain; 2 Comparative Minds Research Group, Department of Psychology, Universitat de Girona, Girona, Catalonia, 17004, Spain

**Keywords:** Wildlife trade, welfare, social responsiveness, personality, behaviour, macaques

## Abstract

Decades of research have illuminated the consequences of early adverse rearing experiences in laboratory macaque populations. However, knowledge of the impact of traumatic episodes in non-laboratory settings remains limited. This study investigates the socio-emotional and behavioural impacts of illegal trade on five macaque species, all victims of poaching. We studied 53 focal subjects residing at the Lao Conservation Trust for Wildlife (LCTW), a former zoo converted into a rescue and rehabilitation centre. We categorised their past experiences into seven aspects, encompassing maternal care and interactions with conspecifics. We assessed social engagement and cooperation by analysing social behaviours and employing the Social Responsiveness Scale. Emotional resilience was evaluated by measuring anxiety levels and the occurrence of abnormal behaviours, supported by a welfare questionnaire. Additionally, the introduction of Cattell’s 16PF questionnaire in macaques for the first time aimed to reveal the influence of traumatic experiences on their personality traits. Our findings underscore the significance of early social exposure to conspecifics. Macaques deprived of juvenile social contact show reduced social behaviours and a tendency towards social avoidance in adulthood. Human-reared macaques display increased abnormal behaviours in social contexts, which compromises welfare. Social deprivation with conspecifics during infancy negatively affects psychological stimulation and overall welfare, with prolonged time in illegal trade correlating with increased anxiety levels. Personality traits, such as ‘Calmness’ and ‘Unfriendliness’, are shaped by rearing conditions, with macaques deprived of social interaction showing higher levels of introversion. In summary, the more time macaques spend in human care with minimal exposure to conspecifics, the more pronounced the impacts on anxiety, abnormal social behaviours, and personality traits, highlighting the significant long-term effects of early rearing conditions on their socio-emotional development.

## Introduction

Macaques have been widely utilised as model organisms in advancing our understanding of various human pathologies, including psychological and neurodevelopmental disorders. ^1^^,^^2^ This preference can be attributed to the substantial commonalities shared between humans and macaques, such as their tendency to live in large social groups, undergo similar developmental stages, and share cognitive and socio-emotional attributes. ^1^^–^^3^ The early years of development represent a particularly vulnerable stage during which high stress exposure or adverse experiences can exert profound influences on brain development. Subsequently, this can lead to deleterious behavioural, cognitive, or emotional outcomes ^4^^,^^5^ (e.g., for review in macaques see Ref. 6).

In humans, “adverse childhood experiences” (ACE) or “children adversity” refer to detrimental environmental experiences during infancy. These experiences encompass physical maltreatment (involving violence, the threat of mistreating, or sexual abuse), emotional abuse (distress), as well as the deprivation of essential inputs such as cognitive and social withdrawal, and neglect. ^7^^,^^8^ These proximal processes during early life are intricately associated with impaired socio-emotional and behavioural capacities in adulthood in both humans, ^9^ and macaques. ^10^^,^^11^ Zhang (2017) ^6^ reviewed the effects of early adverse rearing experiences (EARE), similar to ACE in the context of children, in non-human primates, mostly using Rhesus macaques ( *Macaca mulatta*) as a model species. Many skills and characteristics seem to be affected by EARE. For instance, it is noteworthy that social skills may decline as a result of an increased manifestation of stereotypes, abnormal, and anxiety-like behaviours. ^12^^–^^15^ Similarly, Bellanca and Crockett (2002) ^16^ revealed that the expression and manifestation of abnormal behaviours and stereotypes in pig tailed macaques ( *Macaca nemestrina*) used in invasive research depended on the rearing and housing, being the former condition crucial for locomotor stereotypes exhibition. Lutz and colleagues (2003) ^17^ underpinned these results in captive Rhesus monkeys. The research group found that nurse rearing represented a risk factor for the development of digit-sucking behaviours, whereas, the age at which macaques were individually housed in cages dramatically impacted on the rate of repetitive behaviours, including self-directed stereotypes, self-injury, and self-biting abnormal behaviours. These studies evidenced the critical role of social contact with conspecifics during infancy and juvenility in shaping the typical development of socio-emotional and behavioural skills within macaque species.

In line with this, several studies revealed that macaques housed at research facilities show long-term effects such as cognitive impairment, ^18^^–^^20^ socio-behavioural deficiencies, ^21^ including less play, lower social rank, and an increased frequency of aggressive behaviour. ^22^ Additionally, they may exhibit, impaired sexual behaviour, ^23^^–^^27^ a reduced repertoire of species-typical behaviours, ^28^ maternal neglect and abuse towards offspring, ^29^^–^^31^ and lower social skills in adulthood. ^32^^–^^34^ Furthermore, they can experience psychophysiological impacts, notably the dysregulation of the hypothalamic–pituitary–adrenal axis (e.g., for reviews see Ref. 35).

The capacity of individuals to withstand and recover from these traumatic experiences varies significantly and is influenced by a multitude of factors that include species differences, the age at which the separation from the mother occurs, individual personality traits, and the presence of a secure attachment during infancy. ^11^^,^^36^

Nevertheless, it is important to note, that while early-life adversity can have significant long-term effects on the welfare of macaques, it would be inappropriate to generalise these findings to all laboratory settings. In recent years, there has been increasing recognition of the need for improved welfare standards for primates in research, with more stringent regulations and ethical guidelines now in place to ensure better living conditions and care. ^50^ These advances in animal welfare practices aim to mitigate the negative impacts of early experiences, making it essential to avoid an overly broad characterisation of all laboratory environments as inherently detrimental.

On the top of that, infant and early adolescent trauma may serve as specific vulnerability and mediator factors contributing to several psychological disorders, including post-traumatic stress disorder (PTSD), major depressive episodes, anxiety disorders, cognitive impairment, and emotional dysregulation in both human and macaques. ^1^^,^^2^^,^^37^^,^^38^ In this vein, McLaughlin and colleagues (2019) ^39^ have explored the neurodevelopmental mechanisms that underlie the boundary between adverse childhood experiences in children and psychopathological outcomes in adulthood, including PTSD, major depressive episodes, anxiety, and cognitive impairment. Likewise, Ausderau and colleagues (2023) ^40^ compared the symptoms of depression and anxiety between humans, marmosets, Rhesus, and long-tailed macaques. The vast majority of cited papers on this research focused on severe life experiences and their role in the development of aforementioned disorders, such as maternal separation, ^41^^–^^45^ early social withdrawal, ^46^^,^^47^ and early adverse rearing conditions. ^48^^,^^49^

While extensive literature has been dedicated to the study of early severe experiences in laboratory macaques, ^50^^,^^51^ our understanding of the consequences of such conditions during infancy, juvenility, or throughout the life course in (former) captive macaques, especially those affected by (illegal) trade or poaching, remains limited. This knowledge gap is of paramount importance for the conservation of primates.

According to The IUCN Red List of Threatened Species, long-tailed macaques ( *Macaca fascicularis*) are dramatically threatened by national trade for pets, entertainment purposes for tourists, and social media exploitation. Furthermore, the international trade in this species has burgeoned into a multi-billion-dollar industry, a trend that has increased even more in the wake of the Covid-19 pandemic. ^52^ In the case of the Northern pig-tailed macaques ( *Macaca leonina*), their principal threats in Lao PDR, Vietnam, and Cambodia stem from bushmeat and the pet trade. In Thailand, males of this species are subjected to exploitation, as they are trained for coconut harvesting and may subsequently be sold for up to $1000. ^53^^,^^54^ Similarly, in Lao PDR the primary threats for the stump-tailed macaque ( *Macaca arctoides*) and Assam macaques ( *Macaca assamensis*) include bushmeat, traditional medicine, and the pet trade, being their bones sold in local markets or through social media platforms for the production of glue or balms. ^55^^,^^56^

Globalisation and the prevalence of social media exacerbate this dire situation. For instance, Espinosa & Dias (2016) ^57^ revealed that non-human primates face heightened risks due to interactions with tourists seeking wildlife selfies, thereby contributing to the potential risk of poaching. ^58^ Furthermore, unpublish results declared that a staggering number of over 4,700 long-tailed macaques were offered for sale on Facebook in Indonesia in the years 2020 and 2021 alone (source: IUCN). Meanwhile, the lack of strong wildlife policies in Southeast Asia contributes to this problematic situation. For instance, the Organisation for Economic Co-operation and Development (OECD) underscore in its publication “The Illegal Wildlife Trade in Southeast Asia” (2019) ^59^ the pressing need for effective enforcement of anti-trafficking laws, along with the strengthening of penalties and financial consequences to deter the persisting high-reward, low-risk nature of wildlife trade. As an example, we can highlight “The Wildlife and Aquatic Law 2007” (WAL, 2007) implemented in Lao PDR for the protection of wildlife. This legislation permits the holding of wildlife for breeding and business purposes, criminalises poaching of endangered species, as well as, their illegal trade and commercialisation. Nonetheless, the penalties for violating this law range from imprisonment maximum two years and maximum fine is 600,000 LAK, equivalent to 72 USD. ^60^

Despite these facts, little is known on whether being a victim of the trade along lifespan shapes several socio-emotional and behavioural skills or personality traits and its potential impacts on the quality of life in non-human primates. Lopresti-Goodman and colleagues (2013) ^61^ presented two case studies of rescued chimpanzees from bushmeat and pet trade whose psychological distress—based on abnormal behaviour, stereotypes, social deprivation with conspecifics, hypervigilance, fear, emotional instability and even symptoms of PTSD in adulthood—was linked to abusive experiences in infancy and juvenility and lifelong captivity. Regarding personality traits, Ortín and colleagues (2019) ^62^ found that chimpanzees who experienced social withdrawal during infancy and juvenility, combined with severe abuse, were more likely to display higher levels of anxiety and dominance. In contrast, those who were mother-reared tended to exhibit lower dominance and restraint personality traits than hand-reared individuals.

The use of wildlife by humans is not limited to laboratories and entertainment; practices like breeding and business are inadequately regulated in some regions of Southeast Asia. ^59^ For example, the case of coconut-harvesting pig-tailed macaques, whose psychological well-being was assessed by Schowe and colleagues (2021) ^54^ is emblematic. Having been deprived of social stimulation since infancy or juvenility, enrichment, sensory input, opportunities to exhibit species-typical behaviours, and a high-quality diet, these exploited macaques exhibited a mean welfare score of 4.8±1,2 out of 12 points, indicating an absence of positive mental states and high rates of abnormal behaviours and stereotypes, pointing to symptoms of compromised welfare. ^63^

This research aims to assess the effects of adverse experiences on socio-emotional and behavioural abilities of macaques who are victims of illegal trade. To achieve this, fistly, we studied social responsiveness, affiliative behaviours, and grooming (as positive indicators of psychological well-being), abnormal and anxiety-like behaviours (as negative indicators of psychological well-being), and general welfare and personality traits of the resident macaques at Lao Conservation Trust for Wildlife centre (LCTW). LCTW is a former zoo that has been transformed into an animal rescue and rehabilitation centre, currently housing over one hundred individuals from various macaque species. While many of these macaques were victims of poaching, others were rescued from other forms of exploitation, and in some cases, the details of their past remain unknown. Secondly, we aimed to describe their socio-emotional and behavioural profiles. We considered that the findings of this study may contribute to the understanding of (1) the proximate and (2) ultimate mechanisms involved in socio-emotional development in both human and non-human primates, as well as (3) contribute to the design of more effective and management and rehabilitation procedures for non-human primates in animal rescue and rehabilitation centres.

Building on previous publications, we predicted that adverse and traumatic experiences, including early maternal separation, deprivation of social interactions with conspecifics, undesirable housing, and humanisation, amongst others, may be linked to: (1) impairment of social skills in adulthood, ^21^^–^^31^ (2) difficulties in coping with stress or an increasing expression of stereotypes and abnormal behaviour, indicators of negative welfare ^16^^,^^17^^,^^54^^,^^63^; and (3) the development of specific personality traïts, such as heightened neophobia, increased dominance, or elevated neuroticism. ^62^

## Methods

### Study site and population

Lao Conservation Trust for Wildlife (LCTW) operates in Lao PDR, a key corridor for the illegal trade in Southeast Asia, as a gateway between Thailand, Myanmar, Vietnam, Cambodia, and China. LCTW is registered by the Lao Government (under number 326/MoHA) and in the United Kingdom (under the number 1182501). Since 2018, this organisation has been engaged in the rescue, care, and release of native species victimised by illegal trade, currently providing shelter to over 400 animals across more than 26ha of land. Prior to this date, from 1994 to 2016, this centre was known as “Lao Zoo”, a place where visitors could interact and feed the resident animals, all of which were rescued from illegal trade. The majority of arrivals, both then and now, primarily consist of macaques rescued from the pet trade, where they were found in family settings, temples, or establishments like resorts. Regrettably, relevant information of the rescued macaques was missed between 1994 and 2017, which limits our knowledge of their life experiences. In order to link the background to a lack of social skills, a high expression of abnormal behaviour, a low score of welfare, and certain personality traits, we exclusively selected macaques with meticulously documented life experiences labelling them “focal” individuals. We collected data of abnormal and anxiety-like behaviours defined in the ethogram (Suppl. Tables 3-4 in the extended data), and we administered questionnaires (personality, welfare, social responsiveness) to these focal individuals only. Nonetheless, we gathered social behaviours for both focal and non-focal animals, as social interactions cannot be restricted to certain animals (Suppl. Table 5 in the extended data).

In this study we investigated 53 focal subjects selected from a larger sample of 88 macaques. These focal animals, aged between 1 and 18 years old (mean age ± SD = 8 ± 5 years), were distributed across eleven groups/enclosures (Suppl. Table 1 in the extended data for biographic information). The enclosures for the macaques range in size from 51.72 m ^2^ to 1,236.63 m ^2^, with an average of 594.47 m ^2^. Group sizes vary between 1 and 19 individuals, with an average of 7.53 individuals per group. Across all groups, there are 38 females and 42 males, distributed in different sex ratios. The available space per individual ranges from 7.39 m ^2^ to 109.31 m ^2^, averaging 64.22 m ^2^ per individual, ensuring adequate spatial distribution for each macaque according to group composition ( Table 1).

**Table 1.** Enclosure and group information of focal subjects at Lao Conservation Trust for Wildlife.

Species	Group/Enclosure	Enclosure size (m ^**2**^)	Total individuals	Size per individual (m ^**2**^)	Females	Males	Age range (years)
Stump-tailed macaques ( *Macaca arctoides*)	P1	651,56	7	93,08	3	4	8-21
Stump-tailed macaques ( *Macaca arctoides*)	P2	653,76	8	81,72	4	4	1-15
Rhesus ( *Macaca mulatta*)	P4	949,77	3	63,32	1	2	1-11
Assamese ( *Macaca assamensis*)			1		0	1	
Northern pig-tailed macaques ( *Macaca leonina*)			11		7	4	
Rhesus ( *Macaca mulatta*)	P5	1236,63	8	65,09	4	4	11 months - 11
Assamese ( *Macaca assamensis*)	P5		9		5	4	
Pig-tailed macaque ( *Macaca leonina*)	P5		1		0	1	
Long-tailed macaque ( *Macaca fascicularis*)	P5		1		0	1	
Rhesus ( *Macaca mulatta*)	P6	874,49	3	109,31	1	2	5-10
Assamese ( *Macaca assamensis*)	P6		5		3	2	
Pig-tailed macaques ( *Macaca leonina*)	P7	705,08	7	100,73	4	3	3-8
Stump-tailed macaques ( *Macaca arctoides*)	P8	340,04	5	68,01	3	2	10 months - 17
Stump-tailed macaques ( *Macaca arctoides*)	P9	233,03	5	46,61	3	2	5-8
Assamese ( *Macaca assamensis*)	P10	51,72	2	7,39	0	2	2-12
Long-tailed macaque ( *Macaca fascicularis*)	P10		3		0	3	
Pig-tailed macaques ( *Macaca leonina*)	P10		2		0	2	
Pig-tailed macaques ( *Macaca leonina*)	BP1	50-100	3	16,67-33,33	1	2	7-10
Pig-tailed macaque ( *Macaca leonina*)	BP3	50-100	1	12,5-25,00	1	0	10-13
Assamese ( *Macaca assamensis*)	BP3		3		0	3	

During the course of this research, the composition of macaque groups changed due to the frequent arrival of rescued individuals at the centre. Some of these new arrivals were initially housed separately and gradually introduced to the most compatible group. Others, following unsuccessful introductions, were relocated to quarantine, pending future attempts (see Suppl. Table 2 in the extended data for more details). All the enclosures, except for P10, BP1 and BP3 are naturalised, free ceiling spaces equipped with an electric fence, available wild trees, two holdings for introducing new members and addressing medical issues, a swimming pool, and platforms for the macaques. P10, on the other hand, is a sizable cage with a natural floor that includes platforms, enrichment to hide, four holdings and one swimming pool. The BP enclosures consist of three interconnected 50m ^2^ cages (BP1, BP2 and BP3) that include a concrete floor, swimming pools and one platform each. Resident macaques in BP1 and BP3 share the middle cage BP2, enabling each group to use the additional space in rotational shifts every two days. There are no indoor facilities, and the animals remain outdoors with the whole group, except when necessary for specific reasons, such as medical interventions, cleaning, or repairs. As a consequence of the absence of indoor enclosures, keepers, and staff may enter the enclosures as needed, for tasks such as cleaning, maintenance, or medical procedures. Macaques are fed twice per day with seasonal vegetables, fruits, leaves and seeds, from 9:30 to 10:00 in the morning and from 15:00 to 15:30 in the afternoon.

### Categories

We established several categories with the information collected on ZIMS (Zoological Information Management Software) ^64^ or provided by oral testimonies from LCTW staff about the previous traumatic events of the subjects in order to study which type of early adverse experience or stressful history may impact dramatically on the development of socio-emotional and behavioural skills and personality of the subjects. The information gathered may be incomplete, ambiguous, or scarce, specially of those who arrived at the centre before 2018, which was obtained by former workers (ZIMS) and one-time keepers at Lao Zoo (oral testimonies). Ten categories were created, three of them not being related to the background: sex, current age, and species. Seven categories were referred to the subject’s background: origin, type of rearing, life experience, social exposure during infancy, social exposure during juvenility, mother separation before 14 months old, and age of arrival at the centre (for categories see Suppl. Table 1 in the extended data, for codes’ meaning and details see Table 2).

**Table 2.** Definition of each category and codes.

Category	Code	Meaning	Comments/References
**Sex**	a1	Male	
	a2	Female	
**Estimated Current Age**	b1	More than 0, equal or less than 14 months old	Infants - from 0 to 14 months old
	b2	More than 14 months old, equal or less than 36 months old	Juvenile - from 14 months to 36 months old
	b3	More than 3, equal or less than 8 years old	Adolescence and sexual maturity - 3–8 years old
	b4	More than 8 years old, equal or less than 15 years old	Adulthood - from 8 to 15 years old
	b5	More than 15 years old	Elderly - more than 15 years old ^61^^–^^71^
	b6	Unknown	Estimated by former and current veterinarians according to dentition
**Species**	c1	*Macaca arctoides*	We considered necessary to establish “species” as a category for three reasons
	c2	*Macaca assamensis*	(1) many of the orphans that arrived at the centre in weaning stage were fostered by non-same species surrogate mothers which may impact on their behaviour,
	c3	*Macaca fascicularis*	(2) many groups are mixed species which could influence in the sociability and welfare of the lower-number-species subjects,
	c4	*Macaca leonina*	(3) resilience amongst other crucial behaviours such as conciliation may differ between species ^36^^,^^72^
	c5	*Macaca mulatta*	
**Origin**	d1	Captive conceived	
	d2	Born in the wild	
**Rearing**	e1	Parenting	Reared by parents or surrogate mother or father
	e2	Hand	Reared by humans ^36^^,^^73^
**Life Experience**	f1	Pet	Which usually involves being in chains or cages include orphans
	f2	Entertainment	Working for entertaining tourists such as being caged in resorts, temples, etc.
	f3	Zoo	For those who were born in the former zoo and spend their whole lives in captivity
	f4	Trade	For those whose past is not exhaustively known, but they were rescued from poaching
**Infancy Social Exposure**	g1	Accompanied by conspecifics, more than 80% of infancy	Presence or absence of conspecifics in the subject’s infancy ^74^^–^^76^
	g2	Mixed or accompanied between 80-20% during infancy	Infancy is a period defined from 0 to 14 months old ^77^^–^^80^
	g3	Alone, more than the 80% of infancy	We generated four subcategories, according to the percentage of time that they were exposure to social interactions and conspecifics
	g4	Unknown	
**Juvenility Social Exposure**	h1	Accompanied by conspecifics, more than 80% of juvenility	Presence or absence of conspecifics in the subject’s juvenility
	h2	Mixed or accompanied between 80-20% during juvenility	Juvenility is a stage defined from 14 to 36 months old ^81^^–^^85^
	h3	Alone, more than the 80% of juvenility	We generated four subcategories, according to the percentage of time that they were exposure to social interactions and conspecifics
	h4	Unknown	
**Mother separation**	i1	Yes	This category was established according to the weaning period in rhesus macaques, which is completed at about 10–14 months of age, a period of time in which infant should not be separated from their mothers for normal development ^86^
	i2	No	
**Estimated Age at Arrival**	j1	Equal or more than 0, equal or less than 14 months old	Infants - from 0 to 14 months old
	j2	More than 14 months old, equal or less than 36 months old	Juvenile - from 14 months to 36 months old
	j3	More than 3 years old	Adolescence, adulthood, and elderly ^61^^–^^71^
	j4	Unknown	Estimated by former and current veterinarians according to dentition

### Procedure and data collection

We combined two methods: questionnaires and behavioural observations. Socio-emotional and behavioural skills have been structured in five domains, following the BESSI [Behavioral, Emotional, and Social Skills Inventory] ^87^ proposal, defined in Table 3. The present study has been focused on three of these domains: (1) social engagement, (2) cooperation, and (3) emotional resilience.

**Table 3.** Socio-emotional domains, skills, measures, and procedures during the data collection, following the Behavioural, Emotional, and Social Skills Inventory (BESSI). ^87^

BESSI domain	Domain definition	Socioemotional/behavioural skill	Procedure	Psychological/behavioural measure	Variable/metric
***Social engagement skills***	Capacities used to actively engage with other primates	Sociability	SRS Questionnaire	Social responsiveness	Social responsiveness
		Sociability	16PF Questionnaire	Extraversion	Extraversion
		Leadership skill	Behavioural observations	Dominance hierarchy (aggression-related behaviours)	Elo-rating
***Cooperation skills***	Capacities used to maintain positive social relationships	Social warmth	SRS Questionnaire	Social responsiveness	Social responsiveness
		Social warmth	16PF Questionnaire	Extraversion	Extraversion
				Sensitivity	Sensitivity
				Warmth	Warmth
		Social warmth	Behavioural observations	Other affiliative, other agonistic, social play, socio-sexual and frequency of grooming	Rate of social behaviours
***Emotional resilience skills***	Capacities used to regulate emotions and moods	Resistance to stress	Behavioural observations	Abnormal behaviours	Rate of abnormal
				Self-directed and displacement behaviours	Rate of anxiety
			16PF Questionnaire	Extraversion	Extraversion
				Emotional stability	Emotional stability
			Welfare Questionnaire	Welfare	Positive indicators
					Negative indicators

### Behavioural observations

We collected behavioural data through observations, ^88^ only while macaques had access to their outdoor enclosures, in other words, when they were not in the holding or hospital. Data on macaques’ behaviour was collected from November 14, 2022 to March 22, 2023 (for further information on the collected behaviours, see Suppl. Tables 3-5 in the extended data). We evenly distributed observation sessions of 20 minutes between 6:30 am and 17:00 pm on randomised days (Monday to Sunday). Each troop has been sampled for 12 ± 0.1 hours (min 11.67 hours, max 12.33 hours). Abnormal, anxiety-like, social (affiliative, sexual, agonistic and aggression-related) behaviours were recorded continuously with an all occurrences [multifocal] untimed-event strategy, whereas, the duration and frequency of grooming were recorded with a continuous [multifocal] timed-event strategy. ^89^ The duration of grooming collected will not be used in the present study. For data collection, the observer (first author) used a Sony ICD-PX370 voice recorder in three enclosures (P4, P5, P9) and Zoomonitor software ^90^ in eight enclosures (P1, P2, P6, P7, P8, P10, BP1, BP3), due to a variety of factors such as the lack of visibility, the number of individuals per group and the frequency of behaviours they exhibited. Following a bout recording strategy, we collected behaviours in bouts rather than the single repetition. For instance, in the case of repetitive and odd behaviours, we observed that “hit-self” or “self-bite” behaviours were seldom shown only once, but were performed in a set of repetitions or events. As an example, see Suppl. Video 1 in the extended data, in which an individual (Chock, P9) is exhibiting a “bout” that consists of: float limb, hit-self and self-bite amongst others, such as abnormal behaviours (e.g., self-pinch and abnormal displacement).

### Questionnaires: raters

Three raters were carefully selected based on their substantial experience and significant time dedicated to working with the macaques. The first rater has spent more than four years continuously working with the macaques as a veterinarian and animal management. The second rater had an eight-month period of continuously working with the macaques as an enrichment coordinator. The third rater (first author) engaged with the macaques for a duration of six months, during which she collected the behavioural data for the present research (132 hours observation/total). Raters were explicitly instructed to refrain from discussing their assessments with other participants. We also provided comprehensive guidance on completing the three questionnaires, including discussions and clarifications on concepts associated with animal behaviour and welfare. Finally, we requested raters to respond according to their thoughts and current animal context.

### Social Responsiveness Scale

We used the Social Responsiveness Scale (SRS), previously validated with adults ^91^ and juvenile macaques, ^92^^,^^93^ to evaluate social engagement and cooperation skills. The SRS [short version] scale comprise 14 items, which are associated with statements that need to be scored by a human rater (e.g., “Seems self-confident when interacting with others”) using a Likert rating scale between 1 and 5 (1 = not true 0%, 2 = sometimes true 25%, 3 = often true 50%, 4 = almost always true 75%, and 5 = always true 100%) As described by Balint and colleagues (2021), ^92^ the scoring of the items 1, 5, 7 and 14 were reversed, so that higher scores reflected greater social deficiency for each item.

### Animal Welfare Survey

We employed the Animal Welfare Survey US [AWS] ^94^ to evaluate the emotional and resilience skills domain. This questionnaire consists of 12 items, each one with a statement or a question that needs to be scored or replied by a human rater (e.g., “How often this individual display signs of positive welfare?”) in a Likert rating scale of 1 to 5 (e.g., 1 = never, 2 = rarely, 3 = occasionally, 4 = frequently, 5 = constantly). It includes positive and negative indicators of welfare and well-being, validated with Rhesus macaques, capuchins and chimpanzees ^95^^–^^97^

### Personality questionnaire

We used an adaptation of Cattell’s 16 Personality Factors Questionnaire ^98^^,^^99^ to assess the impacts of traumatic experiences on the development of personality. The questionnaire has been previously administered in chimpanzees ^62^ and comprises 16 items, rated on a Likert scale 1–7. Each item was bipolar and the scores of raters described the subject evaluated closer to one pole or to the other.

## Data analysis

### Behavioural analysis

From the collected data, we calculated the frequency of each behaviour included in the ethogram per individual. Then, we calculated the rate ^89^ for anxiety-like, abnormal and social behaviours (grooming, maternal care, other affiliative, other agonistic, social play and sexual behaviours) per subject based on frequency/observation time. For each group, we create matrices of directed dyadic grooming interactions.

Rank was calculated with the “EloRating” package ^100^ in R, ^101^ considering all dyadic agonistic interactions (dominance and submission) with a winner-loser outcome. Every macaque in each group was assigned a value between 0 (lowest ranking) and 1 (highest ranking). ^102^

### Questionnaires

First, we assessed the interrater reliability of the items of each questionnaire via intra-class correlation coefficients (ICC): ICC (3,1), to evaluate the reliability of individual ratings, and ICC (3,k), which indicates the reliability of mean ratings ^103^ with JASP 0.17.3 software. ^104^ To determine the social responsiveness, animal welfare and personality dimensions, we conducted an Exploratory Factor Analysis using a Robust Unweighted Least Squares (RULS) for factor extraction. ^105^ We applied an orthogonal normalised Equamax rotation to generate uncorrelated factors. ^106^^,^^107^ We based our analysis on polychoric correlations (adequate to Likert-scale ordinal data with asymmetric or with excess of kurtosis data) to achieve factor simplicity and determine factorial structure and goodness of fit. ^106^^–^^109^ We calculated the correction for robust Chi-square with LOSEFER empirical correction. ^110^ We considered factor loadings of the rotated loading matrix as significant when they were 0.5 or higher, in accordance with previous research. ^102^^,^^111^ Finally, we determined the number of factors following two procedures. First, we applied the “latent root criterion” (i.e., eigenvalues above 1) ^92^; and second, we used the optimal implementation of Parallel analysis based on minimum rank factor analysis. ^112^ We assessed the robust goodness of fit using the root-mean-square error of approximation (RMSEA). We considered RMSEA values between.05 and.08 as fair. ^113^ We conducted all the analysis using FACTOR 12.04.01. ^114^

We computed unit-weighted factor scores for each individual, following the procedure described by Weiss and colleagues (2009). ^115^ This calculation involved taking the mean of all the items with salient loadings (>0.5). Items with positive salient loadings were assigned a score of +1 and items with negative salient loadings were assigned a score of -1. Thus, the score for each individual within a particular factor represents the weighted average of that individual’s scores on all the items related to the factor.

### Influence of background on observed behaviours, social responsiveness, welfare, and personality

We assessed the effect of each background-related category on the dependent variables or individual measures using generalised linear models (GLM). We created a total of 11 models, one per each individual measure. As dependent variables we used the rate of (1) social, (2) anxiety and (3) abnormal behaviours, (4) the rank, (5-6) the social responsiveness, (7-9) personality, and (10-11) welfare domains. We included as fixed factors in our full models (a) sex, (b) estimated current age, (c) origin (d) species and background [(e) rearing, (f ) life experience, (g) infancy and (h) juvenile exposure, (i) mother separation, and (j) estimated age at the arrival).

Model interference and the selection of the subsets of best models were performed using dredge function, which is based on the Akaike Information Criterion corrected for small sample sizes (AICc). ^116^ From all models tested, we considered the best explanatory model per each dependent variable those with the lowest AICC or the highest ΔAICc compared to the full model containing all the predictor variables. To assess the collinearity, we examined the value of the variance inflation factor (VIF), with a model considered acceptable when the VIF < 5 between predictor variables. This analysis was conducted in R, ^101^ where we performed GLMs using the “MuMln” package ^117^ and related analysis, including VIF calculations using the “performance” package. ^118^ Plots were generated using the “ggplot2” package. ^119^ An alpha level of 0.05 was used as a cut-off for significance.

## Results

### Behavioural analysis

We divided the range of behaviours into 3 categories: social, abnormal and anxiety-like behaviours. We used the rates for anxiety-like, abnormal, and social behaviours (Suppl. Tables 6-11 in the extended data) to build the individual behavioural profiles.

Social behaviours include grooming interactions (both sender and receiver), social play (involving players regardless of whether they have started the game), other affiliative behaviours such as initiating contact (e.g., eye gaze, touch, following) or reciprocal affiliation (embrace, mutual teeth chattering, mutual touch), other agonistic behaviours (e.g., consolation, requesting/giving support), maternal care for behaviours directed towards unweaned infants, and socio-sexual behaviours for initiators only (Suppl. Table 7 in the extended data). Within social behaviours, it is noteworthy from our results that other agonistic behaviours such as appeasement or consolation, social play, and socio-sexual behaviours were observed at the lowest frequency in the majority of the enclosures, with social play being absent in P1 and P9, and socio-sexual in P6 (Suppl. Table 11 in the extended data). Only two groups exhibited a high rate of social play: P4, which had the highest number of infants and juveniles, and P10. No group exhibited a high rate of sexual behaviours (Suppl. Table 11 in the extended data). Maternal care was naturally observed only in those groups with unweaned infants (P8 and P5).

The anxiety-like category consists of four behaviours: genital self-inspection (including masturbation), scratching/rubbing, others self-directed behaviours, and yawning (Suppl. Table 8 in the extended data). As part of our predictions, we expected to find a high rate of anxiety-like behaviours in all groups. Indeed, our results show that not only was the rate of anxiety-like behaviours high, but also these behaviours were predominant over social and abnormal behaviours in the vast majority of the enclosures (Suppl. Table 10 in the extended data).

Abnormal behaviours were divided into six subcategories due to the wide range of such behaviours included in the ethogram. These subcategories comprised self-directed behaviours (e.g., poke body, grooming stereotypically, self-suck), postural (limited to leg-lift), self-abuse (e.g., self-bite, hit-self, trichotillomania), kinetic (e.g., float limb, pacing, twist), oral (e.g., regurgitation, reingestion, pica), and miscellaneous (e.g., touch urine stream, other abnormal behaviour not included in the ethogram) (Suppl. Table 9 in the extended data). Our findings reveal that the rate of abnormal behaviours was notably high in several groups, being higher than social behaviours in BP3, and slightly lower in P9 and P7 (Suppl. Table 11 in the extended data). All the groups exhibited abnormal behaviour, with the lowest rate observed in P4, which is again the group with more infants and juveniles who therefore arrived at the centre at an early age (Suppl. Table 11 in the extended data).

### Social responsiveness

The reliability of individual ratings (3,1) ranged from 0.29 (Species typical reaction) to 0.76 (Socially tense) with a general mean of 0.52. The reliability of mean ratings (3, k) for the traits ranged from 0.55 (Species typical reaction) to 0.92 (Socially tense) with a mean of 0.75. There were no items with zero or negative values. The inter-rater reliabilities of all 14 items are presented in Suppl. Table 12 in the extended data.

Based on the normed MSA (Measure of Sampling Adequacy) all the items obtained values above 0.5, indicating its adequacy in representing the underlying constructs (Suppl. Table 13 in the extended data). Therefore, we retained all the items in the exploratory factor analysis. Based on the latent root criterion, we identified 2 factors to retain (Suppl. Table 14 in the extended data). The two factors accounted for 73.66% of the variance (Suppl. Table 15 in the extended data). According to the RULS, the value of the Kaiser-Meyer-Olkin (KMO) test was 0.66 (mediocre) [CI 0.364, 0.561] and Bartlett’s Test of Sphericity was significant (B=1755.7; df=91, p<0.001), thus indicating the adequacy of the correlation matrix. RMSEA fit was fair (0.078; [Bootstrap 95% CI 0.055, 0.068]).

According to the latent root criterion and an adequacy load of 0.5, two factors were loaded with the majority of the 14 items (Suppl. Table 14 in the extended data).

We interpreted the load of reverse items (1, 5, 7 and 14) on the factors as negative. Five items positively loaded on the first factor (F1): Socially tense (0.902), Social avoidance (0.875), (Not) Eye contact (0.760), Socially awkward (0.694), and Lonely (0.575). Furthermore, three reverse items scored negatively on this factor, Socially confident (0.885), Playful (0.701), and Communication skills (0.646). This factor was denominated as Social Reluctance. The second factor was labelled as Inappropriate Behaviour, scoring positively with seven items: Bizarre behaviour (0.825), Stereotypes (0.804), Restricted interests (0.784), Stares into space (0.774), Socially awkward (0.691), No physical coordinated (0.656), (Not) Eye contact (0.553); and negatively with Species typical reaction (0.787) and Communication skills (0.627).

### Welfare

The reliability of individual ratings (3,1) ranged from 0.29 (Control of physical environment) to 0.68 (Number of relationships’ satisfaction) with a general mean of 0.47. The reliability of mean ratings (3, k) for the traits ranged from 0.55 (Control of physical environment) to 0.87 (Number of relationships’ satisfaction) with a mean of 0.72. There were no items with zero or negative values. The inter-rater reliabilities of all 12 items are presented in Suppl. Table 16 in the extended data.

Based on the normed MSA (Measure of Sampling Adequacy), all the items obtained values above 0.5 indicating its adequacy in representing the underlying constructs (Suppl. Table 17 in the extended data). Based on the latent root criterion, we identified 2 factors to retain (Suppl. Table 18 in the extended data). The two factors accounted for 76.65% of the variance (Suppl. Table 19 in the extended data). According to the RULS, the value of the Kaiser-Meyer-Olkin (KMO) test was 0.91 (very good) [CI 0.596, 1.200] and Bartlett’s Test of Sphericity was significant (B=1763.4; df=66, p<0.001), thus indicating the adequacy of the correlation matrix. RMSEA fit was close (0.03; [Bootstrap 95% CI 0.049, 0.062]).

According to the latent root criterion and an adequacy load of 0.5, two factors were loaded with the majority of the 12 items (Suppl. Table 20 in the extended data). One single item was loaded in the first factor (F1) Psychological stimulation (0.785); therefore, we labelled this factor Psychological Stimulation. The second factor was positively related to Cope with the stress (0.919), Impact of experiences (0.820), Balance of the experiences (0.707), Control of physical environment (0.560) and Physical health (0.544), and negatively with Negative welfare indicators (-0.886) and Stress frequency (-0.834); and this factor was named Welfare.

### Personality

The reliability of individual ratings (3,1) ranged from 0.005 (Sensitivity/Objectivity) to 0.61 (Social boldness/Shyness) with a general mean of 0.37. The reliability of mean ratings (3, k) for the traits ranged from 0.015 (Sensitivity/Objectivity) to 0.92 (Social boldness/Shyness) with a mean of 0.59. There were no items with zero or negative values. The inter-rater reliabilities of all 16 items are presented in Suppl. Table 21 in the extended data.

Based on the normed MSA (Measure of Sampling Adequacy) three of the items (Sensitivity/Objectivity, Abstractedness/Pragmatism and Perfectionism/Flexibility) obtained values below 0.5 suggesting that they correlated with other items and failing its adequacy in representing the underlying constructs (Suppl. Table 22 in the extended data). Thus, we excluded these items during the exploratory factor analysis. In the second round, all the MSA values were above 0.5 (Suppl. Table 22 in the extended data). Based on the latent root criterion, we identified 3 factors to retain (Suppl. Table 23 in the extended data). The three factors accounted for 76.55% of the variance. According to the RULS, the value of the Kaiser-Meyer-Olkin (KMO) test was 0.833 (good) [CI 0.362, 0.862] and Bartlett’s Test of Sphericity was significant (B=1771.1; df=78, p<0.001), thus indicating the adequacy of the correlation matrix. RMSEA fit was mediocre (0.087; [Bootstrap 95% CI 0.055, 0.073]).

According to the latent root criterion and an adequacy load of 0.5, three factors were loaded with the majority of the 16 items (Suppl. Table 24 in the extended data). Items were previously defined by two adjectives, first one corresponding to the lowest score (1) and second one the highest score (7). We selected the second adjective to label the obtained factors. On the first factor (F1), the items that positively loaded were Pragmatism (0.809), Apathy (0.736) and Conventionalism (0.675), and those that negatively loaded were Unruliness (-0.751) and Openness (-0.610). Thus, we labelled this factor as Introversion. On the second factor (F2), the items positively loading were Self-assurance (0.683), Carelessness (0.563), and Openness (0.529) and the items negatively loading were Flexibility (-0.671) and Shyness (-0.589), thus we named this factor Calmness. Finally, the last factor was related to Detachment (0.792), Cooperation (0.598), Conventionalism (0.587), Emotional unsteadiness (0.559) and Shyness (0.538), and negatively with Affiliation (-0.707), Carelessness (-0.562) and Self-assurance (-0.526); Therefore, this factor was named Unfriendliness.

### Influence of background on observed behaviours, social responsiveness, welfare, and personality traits

Two of the initial categories had to be removed from the analysis, due to the lack of variability amongst the sample: Mother separation (97% of the subjects were separated from their mothers and only 3% were not) and Origin (97% of the subjects were born in the wild, 3% were captive conceived). In the same line, c3 category (Species *-Macaca fascicularis*) was only represented by two subjects, which was not enough data to perform a generalised linear analysis. Therefore, we had to exclude the long-tailed macaques from the GLM analysis, although the description of their socio-behavioural profile is still included in this study (Suppl. Table 6-11 in the extended data). Tables 4 and 5 contain the comparison between the best explanatory model and the full model per each response variable, significant ones being in bold. The “Best Model” was selected according to the lowest AICc. ^120^^,^^121^ The collinearity between the predictors for those best models that seem to be influenced by two variables or more is less than 5 in all cases. Tables 6 and 7 show the best explanatory model per measure, significant predictive variables being in bold.

**Table 4.** Model selection statistics and relative influence of the predictive variables on the variation of the observed behaviours.

Response variables	GLM	Subsets of models	AICc	ΔAICc	VIF
Rate of social behaviours	**Best model 1**	**Juvenile Social Exposure + Sex**	143.27	0.00	≤1.04
	Full model 1	Sex + Life Experience + Species + Rearing + Infancy Social Exposure + Juvenile Social Exposure + Estimated Current Age + Estimated Age at Arrival	187.82	44.52	≥10
Rate of anxiety behaviours	**Best model 2**	**Estimated Age Arrival + Species + Sex**	142.29	0.00	≤1.33
	Full model 2	Sex + Life Experience + Species + Rearing + Infancy Social Exposure + Juvenile Social Exposure + Estimated Current Age + Estimated Age at Arrival	184.63	42.34	≥10
Rate of abnormal	**Best model 3**	**Rearing + Sex**	146.43	0.00	≤1.03
	Full model 3	Sex + Life Experience + Species + Rearing + Infancy Social Exposure + Juvenile Social Exposure + Estimated Current Age + Estimated Age at Arrival	194.25	47.82	≥10
EloRating	**Best model 4**	**Sex**	144.60	0.00	0.00
	Full model 4	Sex + Life Experience + Species + Rearing + Infancy Social Exposure + Juvenile Social Exposure + Estimated Current Age + Estimated Age at Arrival	191.36	46.76	≥10

*Note.* The Best Models in bold have some significant results at *p* < 0.05. AICc = Akaike’s information criterion corrected for small sample sizes. VIF = variance inflation factor. We only select models with a VIF < 5.

**Table 5.** Model selection statistics and relative influence of the predictive variables on the variation of the social responsiveness, personality, and welfare.

Response variables	GLM	Subsets of models	AICc	ΔAICc	VIF
SRS: Social Reluctance	**Best model 5**	**Juvenile Social Exposure**	140.33	0.00	0.00
	Full model 5	Sex + Life Experience + Species + Rearing + Infancy Social Exposure + Juvenile Social Exposure + Estimated Current Age + Estimated Age at Arrival	195.22	54.89	≥10
SRS: Inappropriate Behaviour	**Best model 6**	**Rearing**	142.29	0.00	0.00
	Full model 6	Sex + Life Experience + Species + Rearing + Infancy Social Exposure + Juvenile Social Exposure + Estimated Current Age + Estimated Age at Arrival	196.26	53.97	≥10
16PF: Introversion	**Best model 7**	**Juvenile Social Exposure + Life Experience + Sex+ Species**	306.32	0.00	≤2.12
	Full model 7	Sex + Life Experience + Species + Rearing + Infancy Social Exposure + Juvenile Social Exposure + Estimated Current Age + Estimated Age at Arrival	343.64	37.32	≥10
16PF: Unfriendliness	**Best model 8**	**Rearing**	143.00	0.00	0.00
	Full model 8	Sex + Life Experience + Species + Rearing + Infancy Social Exposure + Juvenile Social Exposure + Estimated Current Age + Estimated Age at Arrival	190.59	47.59	≥10
16PF: Calmness	**Best model 9**	**Rearing**	143.16	0.00	0.00
	Full model 9	Sex + Life Experience + Species + Rearing + Infancy Social Exposure + Juvenile Social Exposure + Estimated Current Age + Estimated Age at Arrival	184.09	40.93	≥10
Welfare: Welfare	**Best model 10**	**Rearing**	314.09	0.00	0.00
	Full model 10	Sex + Life Experience + Species + Rearing + Infancy Social Exposure + Juvenile Social Exposure + Estimated Current Age + Estimated Age at Arrival	363.21	49.12	≥10
Welfare: Psychological Stimulation	**Best model 11**	**Infancy Social Exposure +Life Experience**	101.39	0.00	≤2.02
	Full model 11	Sex + Life Experience + Species + Rearing + Infancy Social Exposure + Juvenile Social Exposure + Estimated Current Age + Estimated Age at Arrival	145.19	43.8	≥10

*Note.* The Best Models in bold have some significant results at *p* < 0.05. AICc = Akaike’s information criterion corrected for small sample sizes. VIF = variance inflation factor. We only select models with a VIF < 5.

**Table 6.** Influence of background on observed behaviours. For each model and predictor, estimates, standard errors (SE), t value, and *p* -values ( *p*).

GLMs	Response variables	Parameters	Estimate	SE	t value	*p*
**Best model 1**	Rate of social behaviour	Intercept	0.45	0.22	2.09	**0.04**
		Juvenile Social Exposure (h2)	0.18	0.35	0.51	0.62
		**Juvenile Social Exposure (h3)**	-0.83	0.31	-2.70	**0.01**
		Juvenile Social Exposure (h4)	-0.56	0.46	-1.22	0.23
		Sex (a2)	-0.44	0.26	-1.68	0.10
**Best model 2**	Rate of anxiety behaviour	Intercept	0.72	0.27	2.69	**0.01**
		Estimated Age Arrival (j2)	-0.37	0.33	-1.12	0.27
		**Estimated Age Arrival (j3)**	0.60	0.30	1.99	**0.05**
		Estimated Age Arrival (j4)	0.90	0.56	1.60	0.12
		**Sex (a2)**	-0.58	0.26	-2.19	**0.03**
		Species (c2)	-0.46	0.34	-1.34	0.19
		**Species (c4)**	-1.14	0.33	-3.50	**0.00**
		**Species (c5)**	-1.48	0.40	-3.72	**0.00**
**Best model 3**	Rate of abnormal behaviour	Intercept	-0.33	0.29	-1.17	0.25
		**Rearing (e2)**	0.67	0.32	2.10	**0.04**
		Sex (a2)	-0.46	0.28	-1.65	0.10
**Best model 4**	EloRating (Rank)	Intercept	0.26	0.17	1.50	0.14
		**Sex (a2)**	-0.66	0.27	-2.39	**0.02**

*Note.* The Best Models in bold have some significant results at *p* < 0.05.

**Table 7.** Influence of background on social responsiveness, welfare, and personality. For each model and predictor, estimates, standard errors (SE), t value, and *p*-values ( *p*).

GLMs	Response variables	Parameters	Estimate	SE	t	*p*
**Best model 5**	SRS: Social Reluctance	Intercept	-0.37	0.20	-1.84	0.07
		Juvenile Social Exposure (h2)	0.07	0.35	0.21	0.83
		**Juvenile Social Exposure (h3)**	1.10	0.30	3.67	**0.00**
		Juvenile Social Exposure (h4)	0.07	0.45	0.17	0.87
**Best model 6**	SRS: Inappropriate Behaviour	Intercept	-0.68	0.27	-2.51	**0.02**
		**Rearing (e2)**	0.89	0.31	2.87	**0.01**
**Best model 7**	16PF: Introversion	Intercept	2.98	1.48	2.02	**0.05**
		Juvenile Social Exposure (h2)	-2.24	1.71	-1.31	0.20
		Juvenile Social Exposure (h3)	2.91	1.54	1.89	0.07
		**Juvenile Social Exposure (h4)**	-5.18	2.33	-2.23	**0.03**
		**Life Experience (f2)**	3.01	1.51	1.99	**0.05**
		Life Experience (f3)	-1.99	2.41	-0.82	0.42
		**Life Experience (f4)**	6.82	2.23	3.05	**0.00**
		**Sex (a2)**	4.79	1.27	3.76	**0.00**
		**Species (c2)**	-4.24	1.65	-2.57	**0.01**
		Species (c4)	-2.62	1.60	-1.64	0.11
		Species (c5)	0.29	2.02	0.14	0.89
**Best model 8**	16PF: Unfriendliness	Intercept	-0.65	0.27	-2.39	**0.02**
		**Rearing (e2)**	0.85	0.31	2.73	**0.01**
**Best model 9**	16PF: Calmness	Intercept	0.64	0.27	2.36	**0.02**
		**Rearing (e2)**	-0.84	0.31	-2.70	**0.01**
**Best model 10**	Welfare: Welfare	Intercept	15.86	1.45	10.91	**0.00**
		**Rearing (e2)**	-5.87	1.66	-3.53	**0.00**
**Best model 11**	Welfare: Psychological Stimulation	Intercept	3.33	0.29	11.46	**0.00**
		**Infancy Social Exposure (g2)**	-0.67	0.37	-1.84	0.07
		**Infancy Social Exposure (g3)**	-0.90	0.32	-2.83	**0.01**
		**Infancy Social Exposure (g4)**	-0.26	0.43	-0.60	0.55
		**Life Experience (f2)**	-0.43	0.22	-1.98	**0.05**
		**Life Experience (f3)**	-0.33	0.41	-0.81	0.42
		**Life Experience (f4)**	-1.28	0.41	-3.14	**0.00**

*Note.* The Best Models in bold have some significant results at *p* < 0.05.

**Figures 1-3.**
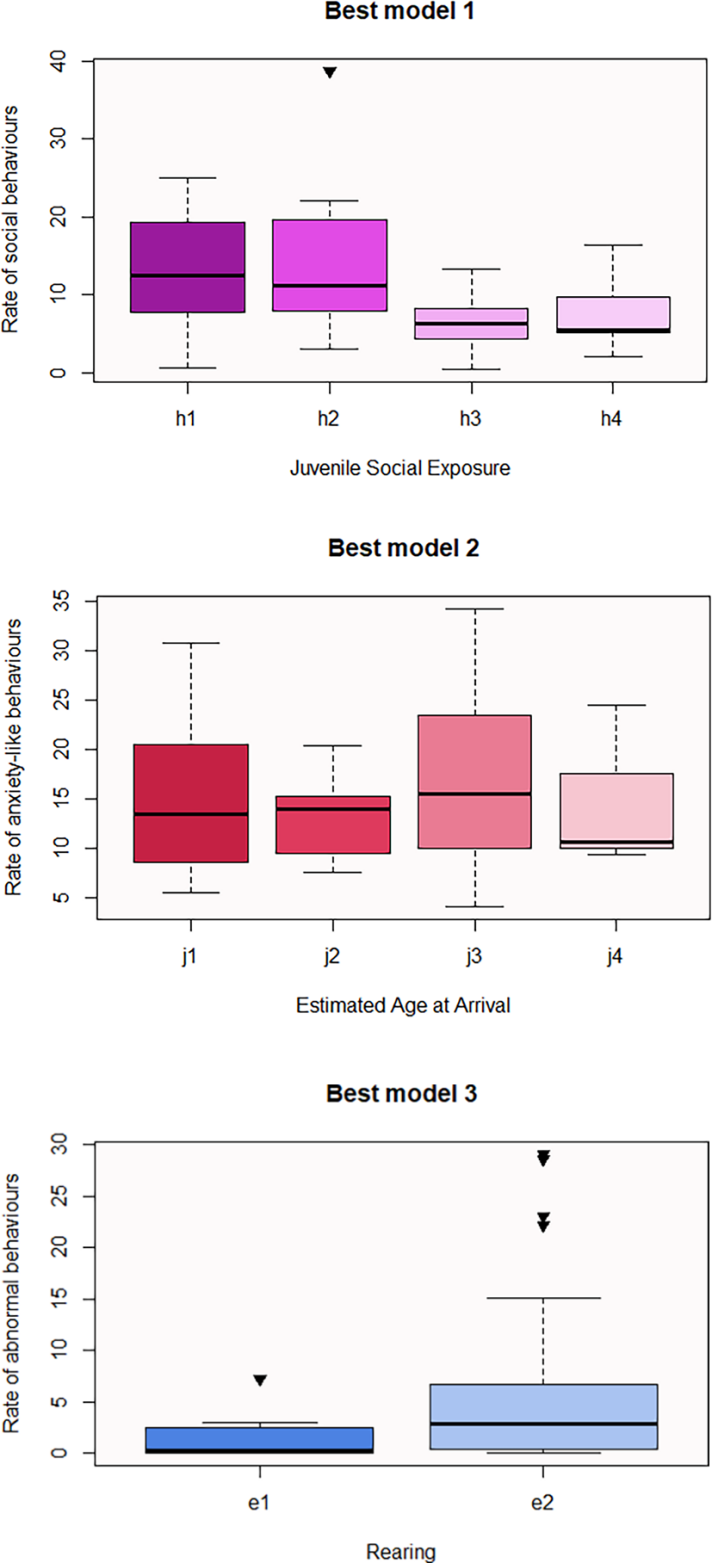
Boxplots representing the influence of background on observed behaviours. Influence of Juvenile Social Exposure on the rate of social behaviours (Best model 1), influence of Estimated Age at Arrival on the rate of anxiety-like behaviours (Best model 2), and influence of Rearing on the rate of abnormal behaviours (Best model 3). Black inverse triangles represent outliers. For definition of the codes see Table 2.

Regarding GLM analysis results for the observed behaviours, the best model that predicts the rate of social behaviours includes Juvenile Social Exposure and Sex, only the first one being significant ( Table 6). As shown in Table 2, the lowest value of this category (h1) corresponds to “Accompanied” whereas, h3 and h4 mean “Alone” and “Unknown” respectively. The direction of the prediction is inverse for h3, therefore subjects who spent their juvenility alone may be less social in the near or later future than those that were accompanied ( Figure 1). Pairwise contrasts of the significant category revealed that h3 or “Alone” subcategory is the predictive variable for lower display of social behaviour (h1-h3 p-value = 0.05; h2-h3 p-value = 0.04). The anxiety-like best model includes Estimated Age at Arrival, Species and Sex ( Table 6). According to this model, the later an individual arrives at the centre (j3), the higher the rate of anxiety behaviours will exhibit (estimate = 0.60) ( Figure 2) (j2-j3 pairwise contrast: p-value = 0.04, estimate = -0.963), males (a1) being more likely to be anxious than females (a2). In addition, the rate of anxiety-like behaviours seems to be significantly lower in rhesus (c5) or pig-tailed macaques(c4) than in stump-tailed (c1), with pairwise contrast being significant in c1-c5 (p-value = 0.003) and c1-c4 (p-value = 0.006) ( Table 6). Third, the best model that predicts the rate of abnormal behaviours consists of Rearing and Sex ( Table 6), with Rearing predictor being the only significant. The direction of e2 predictor’s influence is positive, meaning that macaques that were raised by humans are more likely to exhibit abnormal behaviour than those that were raised by their own parents or by foster parents ( Figure 3). Lastly, Sex is the significant parameter that may predict the acquisition of the rank in the hierarchy ( Table 6). The direction of the influence is negative for a2, meaning that males are more likely to hold a higher rank.

**Figures 4-5.**
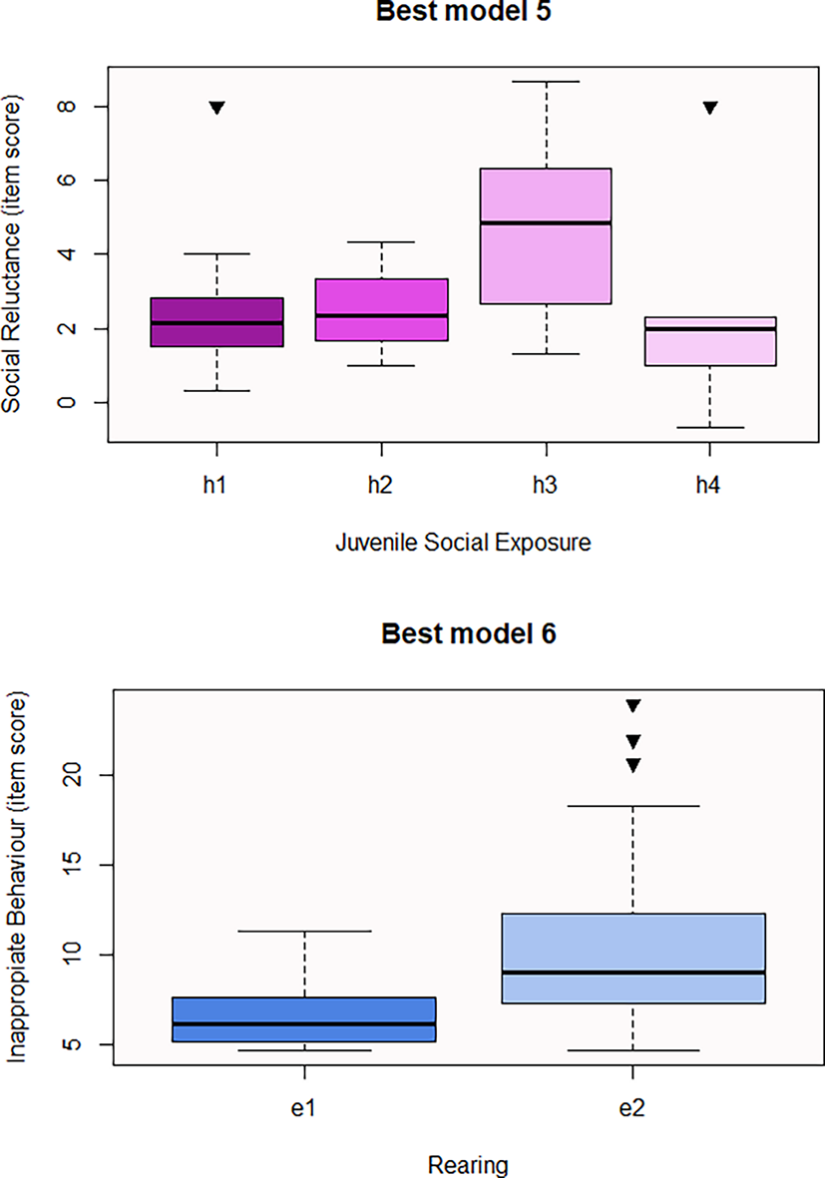
Boxplots representing the influence of background on social responsiveness domains. Influence of Juvenile Social Exposure on Social Reluctance domain (Best model 5) and influence of Rearing on Inappropriate Behaviours domain (Best model 6). Black inverse triangles represent outliers. For definition of the codes see Table 2.

**Figures 6-9.**
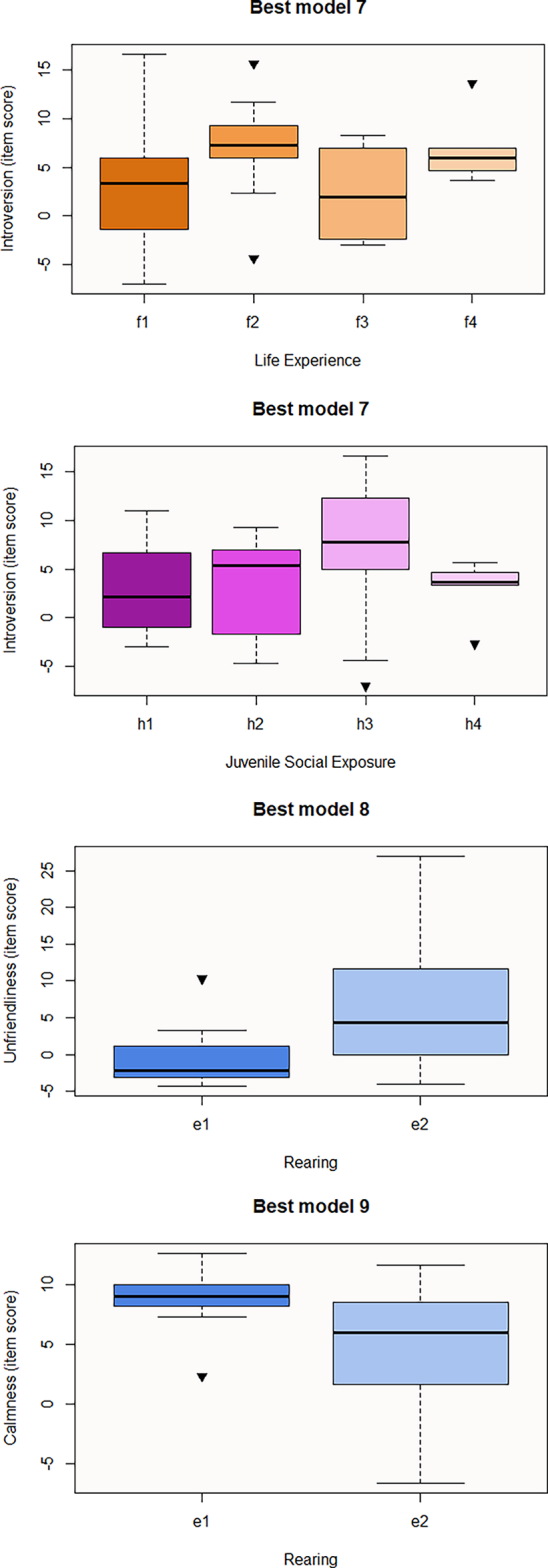
Boxplots representing the influence of background on personality traits domains. Influence of Juvenile Social Exposure and Life Experience on Introversion domain (Best model 7) and influence of Rearing on Unfriendliness (Best model 8) and Calmness (Best model 9) domains. Black inverse triangles represent outliers. For definition of the codes see Table 2.

**Figures 10-12.**
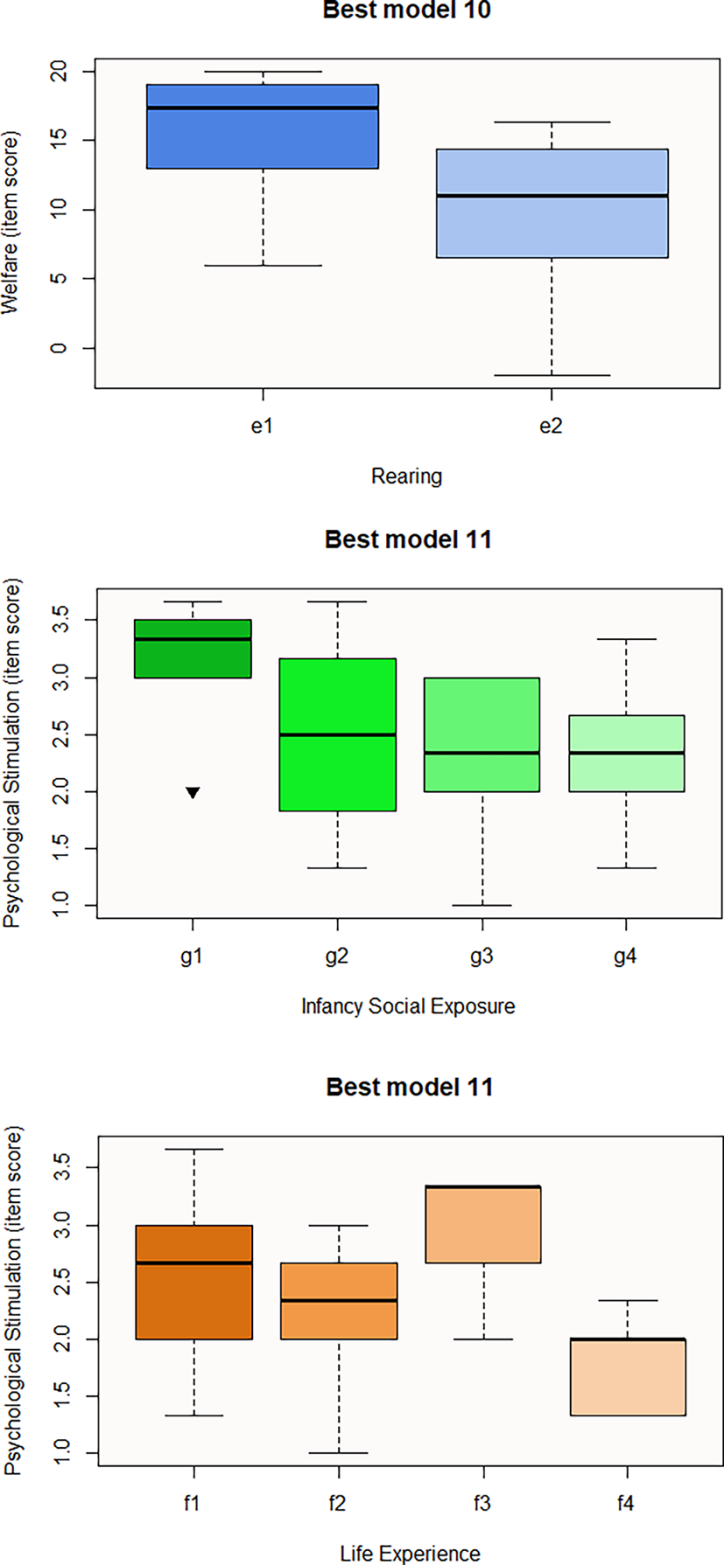
Boxplots representing the influence of background on Welfare domains. Influence of Rearing on Welfare domain (Best model 10), and influence of Infancy Social Exposure and Life Experience on Psychological Stimulation domain (Best model 11). Black inverse triangles represent outliers. For definition of the codes see Table 2.

In relation to the predictive models for the results of the questionnaires, Juvenile Social Exposure is the significant variable that seems to predict the first social responsiveness domain, Social Reluctance ( Figure 4), whereas, Rearing is the predictor for the second domain, Inappropriate Behaviour ( Table 7). Best model 5 consists of Juvenile Social Exposure only, the direction of the influence being positive with h3 and pairwise contrasts being significant for h1-h3 (p-value=0.003) and h2-h3 (p-value=0.03) Therefore, social withdrawal during juvenility may result in higher Social Reluctance in the near or later future. Best model 6 involves Rearing only, with e2 or hand-rearing being the significant variable to predict a higher item score for Inappropriate Behaviour ( Figure 5). Regarding personality questionnaires, we obtained three significant predictive models corresponding to the three personality resulting domains from Cattell 16PF questionnaire ( Table 7). The best explanatory model for Introversion includes Juvenile Social Exposure, Life Experience, Sex and Species. According to GLM results, subcategories f2 (macaques used for human entertainment), f4 (macaques that were rescued from the trade whose past is not exhaustively known), and a2 (females) positively influence this domain ( Figure 6). In contrast, subcategories h4 (unknown juvenile social exposure) and c2 (Assamese macaques) negatively predict this domain, meaning that Assamese tends to be less introverted than the rest of studied species ( Figure 7). However, pairwise contrast shows that within Juvenile Social Exposure, h3 is also a predictive subcategory for lower introversion (h2-h3 estimation=-5.15, p-value=0.007), only f4 would be a significant predictor for this variable (f1-f4 p-value=0.02; f3-f4 p-value=0.04), and any pairwise contrast is significant for Species category. Best Model8, that may predict Unfriendliness, consists of Rearing (e2) whose estimation is positive, meaning that hand-rearing predicts higher item score for this domain ( Figure 8). In contrast, e2 or hand-rearing is the only significant variable that negatively predicts the last personality domain, Calmness ( Table 7 and Figure 9). Regarding the last questionnaire, the best model to predict the Welfare domain consists of Rearing (Best model 10), the influence of e2 predictor being negative for welfare score ( Table 7 and Figure 10). Finally, Best model 11 involves Infancy Social Exposure and Life Experience predictors. Subcategory g3 seems to negatively influence the Psychological Stimulation domain, thus macaques that spent their infancy alone are more likely to be rated lower in welfare than those that were accompanied (g1-g3 p-value = 0.03) ( Table 7) ( Figure 11). Moreover, macaques used for entertainment (f2) and trade (f4) seem to be predictive of the item score for this domain as well ( Figure 12). Nevertheless, pairwise contrast revealed that only f1-f4 comparison is significant at p-value = 0.02 with estimate = 1.276.

## Discussion

The primary objective of this study was to assess the impact of illegal trade on socio-emotional and behavioural skills, ^21^ psychological welfare ^61^^–^^63^ and personality traits ^62^ in former abused macaques. We employed the BESSI framework to describe the influence of our predictor variables on three of the five domains included in this inventory: social engagement, cooperation, and emotional resistance skills.

Firstly, we expected to find socio-behavioural deficiencies in our focal subjects, including reduced play, socio-sexual impairment, lower rank, ^22^^–^^27^ and a reduced repertoire of species-typical behaviours. ^28^ As detailed in Suppl. Table 6 and Table 21 in the extended data, sexual behaviours were rarely exhibited across all enclosures, and social play was predominantly observed in only two groups, as expected. The least exhibited behaviour was “other agonistic” in several groups, which includes the behaviours such as appeasement, giving/asking for support, reconciliation, and consolation. It is worth stressing that we included these behaviours in the “Rate of Social behaviours” category because we considered them to be post-conflict affiliation behaviours, that could be displayed by either the victim or the aggressor. ^122^ Identifying this range of behaviours was challenging due to their species-typical nature and their diversity, and the existence of consolation in macaques is still under debate. ^123^ Thus, we cannot conclusively determine whether agonistic behaviours were genuinely less frequent or if some of them were overlooked during the observation sessions.

### Social engagement and cooperation skills domains: social skills and rank

According to our GLM analysis findings, the rate of social behaviours is significantly influenced by Juvenile Social Exposure ( Table 6). Macaques who experienced social isolation during juvenile years appear to exhibit less social behaviour. Similarly, the social responsiveness domain “Social Reluctance” is influenced by the social withdrawal during juvenility ( Table 7). This domain includes avoidance of social interactions, a lack of social self-confidence, diminished playful interest, and communication skills. Therefore, these results suggest a link between social impairment and social anxiety and the deprivation of social stimulation with conspecifics during the critical period from 14 months to 36 months of age in macaques. For decades, early adverse experiences, such as mother deprivation or infant isolation, have been considered crucial for the development of social skills in non-human primates. ^33^ Our results do not contradict these established facts; instead, they highlight the significance of social stimulation during later developmental stages in shaping social skills, especially in the realms of cooperation (social warmth) and social engagement skills (sociability). Additionally, multiple studies have stressed the critical role of the mother and peers during pre-adolescence in shaping the behavioural profile of primates. ^124^ Likewise, our findings indicate that the Inappropriate Behaviours domain seems to be influenced by the Rearing category, mirroring the predictor models for Welfare and the rate of abnormal behaviours. ^6^ This social responsiveness domain comprises bizarre behaviours and stereotypes amongst other non-typical behaviours of these species. Moreover, it includes items related to communication skills and physical coordination deficiencies. In substance, a high score in this domain means that an individual displays oddly in a social context, closely associated with abnormal behaviour and social anxiety, and indicative of compromised welfare. ^63^

Regarding rank, we did not observe any significant effects related to the background, as predicted. The significant factor that appeared to influence the rank was “Sex”, probably due to the male-dominant nature of the studied macaque species. Bastian and colleagues ^22^ revealed that absence of adults and limited social interactions during early life negatively affect the acquisition of dominance rank, along with age and sex. We suggest that our results differ from previous studies due to the limited background variability in our sample. In the study conducted by Bastian and colleagues, there were three distinct groups with different rearing backgrounds. In contrast, the majority of our subjects have experienced traumatic pasts, and as a result, the predictive strength of the different “background” variables may be significantly lower compared to the influence of Sex.

### Emotional Resilience skills domain: welfare, anxiety, and abnormal behaviours

Secondly, we predicted to find, amongst our sample, psychological distress based on higher rate of anxiety-like behaviours and/or higher expression of abnormal behaviour and stereotypes. ^1^^,^^2^^,^^37^^,^^38^ While the rate of anxiety is notably high across all enclosures, as predicted, our results reveal that the rate of anxiety-behaviours appear to be significantly influenced by the Estimated Age at Arrival, Sex, and Species only ( Table 7). In contrast to previous studies in macaques, bonobos, capuchins, and chimpanzees, ^62^^,^^125^^–^^127^^,^ our findings indicate that males are more likely to exhibit anxiety-like behaviours compared to females. According to our findings, the manifestation of anxiety-like behaviours may vary amongst different species. Despite that the best explanatory model for abnormal behaviours does not include Species category, the rate of both behaviours seems to be significantly lower in Rhesus and pig-tailed compared to stump-tailed or Assamese. This lends support to the idea that using a single species as a model for abnormal or anxiety behaviour within the *Macaca *genus may not be advisable. ^128^

Contrary to our predictions, the social exposure in infancy or juvenility do not appear to predict anxiety in our sample, as reported in other NHP. ^3^ Nonetheless, the j3 subcategory or arriving at the centre in adulthood seems to be significant to predict a higher rate of anxiety. Given that individuals arriving at the centre in later life or adulthood might have spent more time in the illegal trade, we interpreted that the longer an individual has been a victim of the illegal trade, the higher the rate of anxiety behaviours, regardless of the life experience or other conditions. Therefore, we claim that the set of potential distressing events associated with illegal trade, such as exposure to humans, social deprivation with conspecifics, or psychological abuse, have a discernible impact on behavioural outcomes. In essence, elevated levels of anxiety are considerably more prevalent in those macaques who experienced episodes of distress over extended periods, with the absence of peer interactions during juvenility being particularly pivotal for the development of social anxiety.

Regarding the results of the Welfare questionnaire, we named the first domain as “Welfare” because the items that positively loaded on this factor were indicative of preserved welfare, such as “good physical health” and “coping well with the stress”, while those with negative loadings were associated with compromised welfare, such as “high stress frequency”. This is consistent with the traditional use of abnormal behaviour and stereotypes as predictive factors of negative welfare as outlined by Mason and colleagues. ^63^ Several studies have highlighted the profound impact of rearing conditions on the development of behavioural profiles in laboratory macaques, particularly the exhibition of odd repetitive behaviours or stereotypes. ^12^^,^^13^^,^^16^^,^^17^^,^^21^^,^^27^^,^^28^ The best explanatory model for predicting the rate of abnormal behaviours in our sample includes the type of rearing, which aligns with previous findings. In addition, this parameter is also included in the best predictor model for Welfare, as observed in the Inappropriate Behaviour domain. Consequently, hand-rearing conditions emerge as a risk factor for an individual’s inability to cope with stress in social and non-social events, with the resulting detriment to welfare. Since most victims of the illegal trade are separated from their mothers at an early age and reared by humans for purposes such as keeping them as pets or exploiting them for economic purposes, it may be challenging to prevent hand-rearing practices in these circumstances. Nonetheless, rescue centres that frequently receive unweaned rescued primates should consider the possibility of finding foster parents to rear these infants instead of opting for hand-rearing. Similarly, zoos that occasionally care for neglected newborns should contemplate fostering as an alternative to evade the potential effects of hand-rearing or, at the very least, employ both methods to minimise its impact.

Finally, the Psychological Stimulation domain seems to be negatively influenced by the lack of social exposure during infancy and the type of life experiences. EFA analysis revealed that Psychological Stimulation, represented by the item 5 of the Animal Survey Welfare questionnaire, constituted a domain in itself (Suppl. Table 20 in the extended data), suggesting the critical role of psychosocial enrichment in determining welfare in our sample. This item could be rated from “very bored” (1) to “very stimulated” (5), with a higher score indicating positive welfare. Individuals raised in social isolation during infancy for recreational purposes tend to be rated lower in this domain, regardless of the quality of the enrichment. This finding denotes that past experiences can diminish engagement skills of macaques with their environment. Ideally, the past life of the resident animals at zoos and rescue centres should be considered when designing high-quality enrichment protocols to guarantee their welfare.

### Personality traits

Thirdly, we introduced Cattell’s 16PF questionnaire for the first time in macaques, which had previously been validated in humans and chimpanzees. ^62^^,^^98^^,^^99^ We identified three personality domains: the first domain, which we labelled as “Introversion”, showed a positive relationship with pragmatism and apathy, and a negative relationship with openness. The second domain, named “Calmness”, was associated with items opposing vigilance and apprehension. The third factor was designated “Unfriendliness” as it displayed an inverse relationship with affiliation, carelessness and self-assurance. On one hand, we expected to find similar personality traits to those in the reference study, due to the similarity of the backgrounds, despite the macaques being different species. It is important to note that although we did not divide the factor analysis by species due to sample size constraints, species differences could potentially influence the structural outcomes. Given that many of the macaque species in this study were housed in the same facility, we did not expect significant variation. However, future studies with larger sample sizes and separate analyses for each species would be beneficial in exploring these potential effects more thoroughly.

We obtained two opposite and comparable domains (Introversion-Extraversion, Calmness-Anxiety) and one non-related (Unfriendliness) to any of the factors described by Ortin and colleagues. ^62^ Nevertheless, the resulting domains are comparable to those defined by Weiss and colleagues in Rhesus macaques: Dominance, Confidence, Openness (Introversion in our results), Anxiety (Calmness in our results), and Friendliness (Unfriendliness in our results). ^129^ On the other hand, we predicted that the personality profiles may be impacted by, at least, one of our background categories, as occurred in the baseline study. ^62^ GLM analysis shows that the Introversion factor seems to be impacted by several predictive variables which were both related (Juvenile Social Exposure and Life Experience) and not related to the background (Sex and Species) ( Table 7).

Regarding Sex, females tend to be more introverted than males, which aligns with macaques’ social structure, where males have to leave their natal group and socialise to be integrated in other groups, while females typically remain in their original group. ^130^ Regarding Species, it is worth recalling that most of the groups are mixed-species, except for P1, P2, P8, P9 (stump-tailed only) and BP1 (three pig-tailed only). Because of that, we cannot be certain whether this influence is accurate or caused by the unequal composition of the group. Furthermore, pairwise contrast reveals that the difference between species is not significant enough to predict the introversion trait in individuals. We suggest conducting additional research to thoroughly investigate macaque personality at the species-level.

The variables related to the background that predict Introversion are j4 or unknown juvenile social exposure, f2 or entertainment life history and f4 or unknown past in illegal trade. At first sight, we could only take into account f2, which means that those macaques that were used or exploited for human entertainment are more likely to be introverted than those that were pets or born in the zoo. Nevertheless, those individuals who were raised in social isolation during juvenility are more likely to be introverted than those who were accompanied, according to the pairwise contrast analysis. We consider these significant results to be in line with our reference study in terms of a reduced social interaction in early life shapes extroversion-introversion traits in individuals. ^62^ Finally, the best explanatory models for both Calmness and Unfriendliness domains are predicted by the same subcategory, e2, in opposite directions: hand-rearing individuals are more prone to be more anxious and unfriendly or less calm and friendly. We assert that individuals’ anxiety levels and social warmth may also be related to early mother separation and high exposure to humans in early life as this is what being hand-reared implies. ^61^^,^^62^ These findings are consistent with previous research in NHP. ^3^^,^^6^^,^^20^^,^^21^^,^^42^^,^^48^^,^^131^

Consequently, the personality structure of the study sample seems to be shaped by the adverse past according to our results. Our results show potential for the use of Cattell’s 16PF for the assessment of personality in macaques. Nonetheless, as shown in Suppl. Table 21 in the extended data, three of the items (Sensitivity-Objectivity, Abstractedness-Pragmatism and Perfectionism-Flexibility) obtained ICC 3,k values below 0.5, which indicates poor reliability. Whilst we obtained significant results consistent with our predictions, raters agreed with (1) the complexity of the adjectives to describe the personality traits of the focal macaques, (2) the need to have a deeper understanding of the focal subjects and (3) macaque behaviour to fill the questionnaires. Therefore, further studies on the use of Cattell’s 16PF questionnaire in personality assessment should be conducted to evaluate its suitability in macaques. We also suggest using a simpler and shorter questionnaire validated in *Macaca fuscata* ^132^ or commonly implemented and validated questionnaires in macaques as the Hominoid Personality Questionnaire. ^129^

Overall, these results would be in concordance with (1) the baseline study of Lopresti-Goodman in rescue chimpanzees, ^61^ which states that victims of the wildlife trade tend to exhibit psychological distress and more stereotype; (2) former research in bushmeat chimpanzees, ^62^ which states that traumatic past predicts higher anxiety in the victims, and (3) in exploited macaques ( *Macaca leonina*), ^54^ which states that stressful episodes related to the use and abuse of macaques for economic profits leads to their detrimental welfare.

## Conclusion

In conclusion, we have determined that early adverse experiences related to illegal trade exert a significant and lasting impact on the development of social, emotional, and behavioural skills, as well as personality traits in *Macaca arctoides, Macaca assamensis, Macaca leonina* and *Macaca mulatta.* Notably, the absence of social stimulation during the juvenile phase (14-36 months) predicts a reduced rate of social behaviours, increased social avoidance in both early and later life, and higher levels of introversion. Hand-rearing also plays a pivotal role in shaping sociability and social warmth, serving as a strong predictor for the exhibition of inappropriate behaviours in social contexts. Furthermore, being raised by humans impacts the development of resistance to stress and emotional resilience skills, correlating with a higher rate of abnormal behaviour and compromised welfare. Additionally, hand-rearing seems to significantly influence personality traits, especially leading to elevated scores in unfriendliness and anxiety. The use of macaques for human entertainment emerges as the life experience that most profoundly affects the welfare score and the manifestation of introversion traits. Finally, macaques deprived of social exposure during infancy tend to exhibit lower skills of social and environmental engagement, contributing to detrimental welfare.

We propose three avenues for future research (1) further comparative studies to clarify the differences between diametrically opposite backgrounds (e.g., laboratory macaques *versus* former pet macaques *versus* species-typical rearing macaques), and their impacts on behavioural, emotional and social skills; (2) additional research into the application and effectiveness of Cattell’s 16PF questionnaire in macaques; and (3) more pragmatic studies on primates who were victims of illegal trade.

As a future prospect, we hope that this and further studies on whether prolonged traumatic experiences impact on socio-emotional and behavioural skills, may serve to the conservationist struggle against illegal trade. For instance, proving the severe repercussions of trafficking on primates could contribute to the strengthening of laws and policies aimed at wildlife protection, while simultaneously bolstering penalties and fostering public education to discourage this practice.

## Author contributions

**AR**: Conceptualization, data curation, formal analysis, investigation, methodology, software, visualisation, writing—original draft preparation; **MP**: Formal analysis, methodology, supervision, writing—review & editing; **ML**: Conceptualization, formal analysis, methodology, project administration, resources, supervision, writing—review & editing.

## Ethics statement

The study was based purely on observational data without any invasive interventions. It was conducted in accordance with all national and institutional guidelines for the care and management of primates as established by each of the collaborating institutions, Association for the Study of Animal Behaviour/Animal Behavior Society and the Spanish Government (RD/53/2013).

## Data Availability

**OSF:** Impacts of illegal trade on socio-emotional and behavioural skills in macaques, https://doi.org/10.17605/OSF.IO/RZVMY. ^133^ The project contains the following underlying data:
- Influence of background GLM Models.R - Rawdata-Rdatabase.xlsx **OSF**: Impacts of illegal trade on socio-emotional and behavioural skills in macaques, https://doi.org/10.17605/OSF.IO/3QZ7P. ^133^ This project contains the following extended data:
• Supplementary Table 1.docx (Biographic information of focal subjects). • Supplementary Table 2.docx (Group composition’s change during the observation period). • Supplementary Table 3.docx (Catalogue of abnormal behaviours). • Supplementary Table 4.docx (Catalogue of anxiety-like behaviours). • Supplementary Table 5.docx (Catalogue of social behaviours). • Supplementary Table 6.docx (Rate for social, abnormal and anxiety-like behaviours). • Supplementary Table 7.docx (Rate for grooming interaction and social behaviours: maternal care, other affiliative, other agonistic, social play and socio-sexual). • Supplementary Table 8.docx (Rate for anxiety-like behaviours: genial self-inspection, other self-directed, scratching/rubbing, self-groom and yawning). • Supplementary Table 9.docx (Rate for abnormal behaviours). • Supplementary Table 10.docx (Average rate of observed behaviours (social, abnormal, anxiety-like) per enclosure). • Supplementary Table 11.docx (Mean inter-rater reliability of 14 social responsiveness scale questionnaire items for focal macaques at LCTW). • Supplementary Table 12.docx (Mean inter-rater reliability of 14 social responsiveness scale questionnaire items for focal macaques at LCTW). • Supplementary Table 13.docx (Values of the normed Measure of Sampling Adequacy for the SRS items according to the RULS). • Supplementary Table 14.docx (The two factors extracted with latent root criterion in the SRS questionnaire). • Supplementary Table 15.docx (Explained variance based on eigenvalues for the SRS). • Supplementary Table 16.docx (Mean inter‐rater reliability of 12 welfare questionnaire items for focal macaques at LCTW). • Supplementary Table 17.docx (Values of the normed Measure of Sampling Adequacy for the Welfare items according to the RULS). • Supplementary Table 18.docx (The two factors extracted with latent root criterion in the Welfare questionnaire). • Supplementary Table 19.docx (Explained variance based on eigenvalues for the Welfare questionnaire). • Supplementary Table 20.docx (The two factors extracted with latent root criterion in the Welfare questionnaire). • Supplementary Table 21.docx (Mean inter‐rater reliability of 16 personality questionnaire items for focal macaques at LCTW). • Supplementary Table 22.docx (Values of the normed Measure of Sampling Adequacy for the 16PF items according to the RULS during the first round (16 items) and the second round (without items with MSA below 0.5). • Supplementary Table 23.docx (Explained variance based on eigenvalues for the 16PF). • Supplementary Table 24.docx (The three factors extracted with latent root criterion in the 16PF questionnaire). • Supplementary Video. mp4 (Example of a sequence of several bouts of abnormal behaviour). Data are available under the terms of the Creative Commons CC BY Attribution 4.0 International (CC-BY 4.0).
