# Supplementary material for: Impacts of illegal trade on socio-emotional and behavioural skills in macaques
Source: F1000Res. 2024 Nov 29;13:188. Originally published 2024 Mar 11. [Version 2] doi: 10.12688/f1000research.144232.2 (PMC11384201; doi:10.12688/f1000research.144232.2)
Supplement: Peer review history

# Reviewer report: Reviewer response for version 2

**DOI:** 10.5256/f1000research.174729.r348038

**Published:** 2024-12-31

**Authors:** Sam Shanee

**Affiliations:** 1 Neotropical Primate Conservation,, Windrush, UK

This paper provides interesting results on the long term effects of previous illegal captivity on behavior in rehabilitated macaques. It is an important addition to the literature on this subject and provides further evidence (If needed!) that the practice of primate pet keeping is cruel and damaging. The paper is nearly ready for indexing in my opinion, but I suggest a proof edit by the journal or other academic to improve style more than anything. 

 Specific points: It would be better to refer to Stereotypes as stereotypy, as the first is not always the correct term. 

 The Introduction is overly long and wordy, it could be easily reduced by rephrasing (instead of so and so (20XX) found that...., the use of the more standard Primates have been found to....(so and so 20XX) is better.

 The second half of the first paragraph of methods (About missing data, would be better placed at the end of the categories section.

 Questionnaires raters section would be better coming after the personality questionnaire section.

 The first one and half paragraphs of the results (To just after where suppl table 7 is mentioned), is actually methods, as is the first part of results paragraph 4 (to mention of suppl table 9).

 There is at least one paragraph made of single sentence, this needs to be combined into one of the other paragraphs. Also the continued use of words such as  'for instance, furthermore, likewise, on top of that, etc' at the start of many sentences can be removed (The English and writing in general should be polished before publication).

 Other minor details to look out for include:

 Add the genus at first mention of Macaques (start of intro).

 Children adversity should be childhood adversity

  In the behavioral observation section it states that data was only collected when outdoors, whereas previously the methods state that there are no indoor facilities, hence this is redundant. Similarly, in the Welfare section it states 'One single item was loaded in the first factor (F1) Psychological stimulation (0.785); therefore, we labelled this factor Psychological Stimulation' the second half of this sentence is redundant. A careful proof reading would uncover other inconsistencies.

Is the work clearly and accurately presented and does it cite the current literature?

Yes

If applicable, is the statistical analysis and its interpretation appropriate?

I cannot comment. A qualified statistician is required.

Are all the source data underlying the results available to ensure full reproducibility?

Yes

Is the study design appropriate and is the work technically sound?

Yes

Are the conclusions drawn adequately supported by the results?

Yes

Are sufficient details of methods and analysis provided to allow replication by others?

Yes

Reviewer Expertise:

I am a primatologist, an academic, and conservationist working in applied field research in the neotropics for almost 20 years.

I confirm that I have read this submission and believe that I have an appropriate level of expertise to confirm that it is of an acceptable scientific standard.

# Reviewer report: Reviewer response for version 2

**DOI:** 10.5256/f1000research.174729.r344467

**Published:** 2024-12-27

**Authors:** Lief Erikson Gamalo

**Affiliations:** 1 University of the Philippines Mindanao, Davao City, Philippines

The author have already revised the manuscript based on the comments of the reviewers. With this in mind, I approved the indexing of this paper. Thank you very much.

Is the work clearly and accurately presented and does it cite the current literature?

Yes

If applicable, is the statistical analysis and its interpretation appropriate?

I cannot comment. A qualified statistician is required.

Are all the source data underlying the results available to ensure full reproducibility?

Yes

Is the study design appropriate and is the work technically sound?

Yes

Are the conclusions drawn adequately supported by the results?

Yes

Are sufficient details of methods and analysis provided to allow replication by others?

Yes

Reviewer Expertise:

NA

I confirm that I have read this submission and believe that I have an appropriate level of expertise to confirm that it is of an acceptable scientific standard.

# Reviewer report: Reviewer response for version 1

**DOI:** 10.5256/f1000research.157991.r267641

**Published:** 2024-09-09

**Authors:** Lauren M. Robinson

**Affiliations:** 1 Mount Holyoke College (Ringgold ID: 7397), South Hadley, Massachusetts, USA

**Abstract:**

 Clear and very easy to follow

**Introduction**

 Overall, I find this to be well written with lots of great citations. My specific comments are as follows

 - These paragraphs are way too long, they need to be broken up for readability.

 - “In humans…” is a good place to break it up.

 - “In line with this…” is another good place. This paragraph can easily be broken into three.

 - “ multitude of factors, that include” delete comma

 - Delete “On top of that” as opening of next paragraph

 - ”being a victim of the trade along lifespan”, I don’t understand what this means. Across lifespan perhaps?

 - ”The use of wildlife”, paragraph break here

 - ”For all of that”, this is somehow too informal and confusing. All of what? What you just reviewed? I’d pick a new start to this paragraph.

 - ”Currently, LCTW houses more than one hundred individuals from various species of macaques, which were victims of poaching.” Is every animal there the victim of poaching? This sentence is confusing.

 - ” Furthermore, we considered”, delete furthermore

 - ”traumatic experiences including mother separation” comma between experiences and including

 - I like the predictions, they make sense. Though, development of these traits, doesn’t everyone have some sort of development? Would you be predicting an increased development or the like? It’s fine but a better wording wouldn’t hurt.

**Methods**

 - Lots of great details here, very clear methods

 - I couldn’t find the supplementary table but I’m glad you have it as the writing is clear but it’s a huge chunk of text to take in. If there’s a cleaner way to do it with the table included, I’d recommend considering it.

 - ”three of them not being” instead of current word order

**Results**

 - Very clear tables, easy to understand

 - I think the explained variance could probably go in supplementary as saying what was the highest is enough and this could save some space on a long paper

 - If I’m understanding correctly, you’re using 5 species in all of your structural work. How do you factor that in given the differences between macaque species? I see you accounted for it in the later analysis but it still could influence structure and thus scores before you get that far. I understand why you did it with that sample size but just want to understand how you considered its potential impact on your structures.

 - Very thorough statistics work

 - Boxplots are nice

 - Very interesting results

**Discussion**

 - I like the discussion. It doesn’t go beyond the data but the work adds nicely to the existing research

 - I’d break up the personality paragraph, it’s quite long

 -”We suggest conducting additional research to thoroughly investigate macaque personality at the species-level”, yes this makes good sense

 - I like the suggestions for the future, very sensible

Is the work clearly and accurately presented and does it cite the current literature?

Yes

If applicable, is the statistical analysis and its interpretation appropriate?

Yes

Are all the source data underlying the results available to ensure full reproducibility?

Yes

Is the study design appropriate and is the work technically sound?

Yes

Are the conclusions drawn adequately supported by the results?

Yes

Are sufficient details of methods and analysis provided to allow replication by others?

Yes

Reviewer Expertise:

Animal welfare, cognition, personality

I confirm that I have read this submission and believe that I have an appropriate level of expertise to confirm that it is of an acceptable scientific standard.

# Response

**Published:** 2024-11-11

**Authors:** Miquel Llorente

**Affiliations:** Psychology, University of Girona, Girona, Catalonia, Spain

**Abstract:**

 Clear and very easy to follow

**Thank you for your feedback.**

**Introduction**

 Overall, I find this to be well written with lots of great citations. My specific comments are as follows

 - These paragraphs are way too long, they need to be broken up for readability.

 - “In humans…” is a good place to break it up.

 - “In line with this…” is another good place. This paragraph can easily be broken into three.

**Thank you for your valuable feedback. We have reviewed the introduction and made adjustments to improve its readability. Specifically, we have shortened and broken up some of the longer paragraphs to enhance the flow and clarity of the text. We believe these changes will make the section more accessible for readers.**

 - “ multitude of factors, that include” delete comma

**The deleted the comma.**

 - Delete “On top of that” as opening of next paragraph

**We deleted it.**

 - ”being a victim of the trade along lifespan”, I don’t understand what this means. Across lifespan perhaps?

**Thank you for pointing that out. We have revised the phrase "across the lifespan" to "throughout the life course" to provide clearer meaning and better align with the context. We hope this addresses your concern.**

 - ”The use of wildlife”, paragraph break here

**Thank you, we broke the paragraph here.**

 - ”For all of that”, this is somehow too informal and confusing. All of what? What you just reviewed? I’d pick a new start to this paragraph.

**We deleted this.**

 - ”Currently, LCTW houses more than one hundred individuals from various species of macaques, which were victims of poaching.” Is every animal there the victim of poaching? This sentence is confusing.

**Thank you for your comment. We have revised the sentence to clarify that while most of the macaques at LCTW are victims of poaching, some were rescued from other forms of exploitation, and in some cases, the details of their past are not known. We hope this revision helps to address the confusion.**

 - ” Furthermore, we considered”, delete furthermore

**We deleted “Furthermore”.**

 - ”traumatic experiences including mother separation” comma between experiences and including

**We included the comma.**

 - I like the predictions, they make sense. Though, development of these traits, doesn’t everyone have some sort of development? Would you be predicting an increased development or the like? It’s fine but a better wording wouldn’t hurt.

**Thank you for your insightful comment. We have revised the wording to clarify that we are predicting an increased development of certain traits, such as heightened neophobia, dominance, or neuroticism, as a result of adverse experiences. We hope this revised phrasing better conveys the intended meaning.**

**Methods**

 - Lots of great details here, very clear methods

**Thanks for your comment.**

 - I couldn’t find the supplementary table but I’m glad you have it as the writing is clear but it’s a huge chunk of text to take in. If there’s a cleaner way to do it with the table included, I’d recommend considering it.

**Thank you for your feedback. The supplementary table is indeed included, and you can find the link to access all materials at the end of the article. We hope this will provide a clearer and more accessible way to review the information.**

 - ”three of them not being” instead of current word order

**Thank you, we replaced it.**

**Results**

 - Very clear tables, easy to understand

**Thank you for your feedback.**

 - I think the explained variance could probably go in supplementary as saying what was the highest is enough and this could save some space on a long paper

**Thank you for your suggestion. We have moved all the tables containing the details of the ICC values and the factor analyses to the supplementary materials, which should help save space in the main paper while still providing all the necessary information.**

 - If I’m understanding correctly, you’re using 5 species in all of your structural work. How do you factor that in given the differences between macaque species? I see you accounted for it in the later analysis but it still could influence structure and thus scores before you get that far. I understand why you did it with that sample size but just want to understand how you considered its potential impact on your structures.

**Thank you for your insightful comment. We did not divide the factor analysis by species due to the sample size. We anticipated that there would not be significant differences across species, especially since many of the species were housed in the same facility. However, we acknowledge the potential impact of species differences on the structural findings. This is a valuable point, and we will include it in the discussion section as a consideration for future research: “ *On one hand, we expected to find similar personality traits to those in the reference study, due to the similarity of the backgrounds, despite the macaques being different species.*** ***It is important to note that although we did not divide the factor analysis by species due to sample size constraints, species differences could potentially influence the structural outcomes. Given that many of the macaque species in this study were housed in the same facility, we did not expect significant variation. However, future studies with larger sample sizes and separate analyses for each species would be beneficial in exploring these potential effects more thoroughly*** **.“**

 - Very thorough statistics work

**Thank you for the feedback.**

 - Boxplots are nice

**Thank you for the feedback.**

 - Very interesting results

**Thank you for the feedback.**

**Discussion**

 - I like the discussion. It doesn’t go beyond the data but the work adds nicely to the existing research

**Thank you for the feedback.**

 - I’d break up the personality paragraph, it’s quite long

**Thank you for the suggestion. We broke it.**

 -”We suggest conducting additional research to thoroughly investigate macaque personality at the species-level”, yes this makes good sense

**Thank you for the feedback.**

 - I like the suggestions for the future, very sensible.

**Thank you for the feedback.**

# Reviewer report: Reviewer response for version 1

**DOI:** 10.5256/f1000research.157991.r313260

**Published:** 2024-08-29

**Authors:** Augusto Vitale

**Affiliations:** 1 Istituto Superiore di Sanità, Rome, Italy

This is a very interesting and important piece of work. I am not in the field of conservation, but it looks like many of the conclusions would be simply common sense. However, often, science simply demonstrates the obvious with the support of scientific data.

 The authors have achieved exaclty that: supporting important and "common sense" conclusions" with an impressive quantity of results obtained adopting a strictly scientific methodology.

 As I said, I am not in the field of conservation, but I suppose this article will become an important point of reference for the professionals involved in this specific field of primatology.

 I have a concern about the paper, and some stylistic suggestions.

 My concern is that the first part of the Introduction must be significantly shortened. It gives the impression that the paper is about the welfare of monkeys in the lab, which is not at all. It is also confusing: it reads both like a report on how lab research has demonstrated the importance of the early -life trauma in the future development of adult behaviour, as well as a generalised and vague accusation of mis-treatment of primates used in lab reserarch. To a not specialised reader not familiar with primate lab research, gives the imression that primates in labs are inevitably subject to abuse and trauma. This is an unfair generalisation. Critical cases do exist, but the science of welfare of primates housed in research labs has made significant progress which cannot be simply dismissed. I strongly advice the authors to significantly cut the first part of the Introduction, and re-focus it.

 Beyond that, probably I would suggest to semplify the tables and present the numbers of the statistics in a supplementary part. Furthermore, do not refer to Table 1 for explanations that can be directly inserted in the text.

 I congratulate with the authors for this impressive and important research.

Is the work clearly and accurately presented and does it cite the current literature?

Yes

If applicable, is the statistical analysis and its interpretation appropriate?

I cannot comment. A qualified statistician is required.

Are all the source data underlying the results available to ensure full reproducibility?

Yes

Is the study design appropriate and is the work technically sound?

Yes

Are the conclusions drawn adequately supported by the results?

Yes

Are sufficient details of methods and analysis provided to allow replication by others?

Yes

Reviewer Expertise:

ethology, ethics of research, animal experimentation

I confirm that I have read this submission and believe that I have an appropriate level of expertise to confirm that it is of an acceptable scientific standard, however I have significant reservations, as outlined above.

# Response

**Published:** 2024-11-11

**Authors:** Miquel Llorente

**Affiliations:** Psychology, University of Girona, Girona, Catalonia, Spain

This is a very interesting and important piece of work. I am not in the field of conservation, but it looks like many of the conclusions would be simply common sense. However, often, science simply demonstrates the obvious with the support of scientific data.

 The authors have achieved exactly that: supporting important and "common sense" conclusions" with an impressive quantity of results obtained adopting a strictly scientific methodology.

 Response: Thank you very much for your kind words and for your thoughtful comments. We appreciate your perspective on the conclusions, and we fully agree that many of them may seem like common sense. However, we believe that common sense alone does not allow for the level of precision and detail required to fully understand the impact of such practices on animal welfare and the socio-emotional development of individuals. Through the scientific approach we adopted, we have been able to quantify and rigorously assess these effects, providing data that would otherwise be difficult to obtain or appreciate. For that, we suppose this article will become an important point of reference for the professionals involved in welfare and conservation of primates.

 I have a concern about the paper, and some stylistic suggestions.

 My concern is that the first part of the Introduction must be significantly shortened. It gives the impression that the paper is about the welfare of monkeys in the lab, which is not at all. It is also confusing: it reads both like a report on how lab research has demonstrated the importance of the early -life trauma in the future development of adult behaviour, as well as a generalised and vague accusation of mis-treatment of primates used in lab reserarch. To a not specialised reader not familiar with primate lab research, gives the imression that primates in labs are inevitably subject to abuse and trauma. This is an unfair generalisation. Critical cases do exist, but the science of welfare of primates housed in research labs has made significant progress which cannot be simply dismissed. I strongly advice the authors to significantly cut the first part of the Introduction, and re-focus it.

 Response: Thank you for your thoughtful feedback. We fully understand your concern about the first part of the introduction potentially being misinterpreted as focusing primarily on the welfare of macaques in lab settings, and we appreciate the suggestion to shorten it. Our intention was to provide context for the broader issue of early-life trauma in macaques, particularly in relation to the detrimental impacts of illegal trade and poaching, which are less well understood. The references to early adverse rearing experiences were meant to illustrate the continuum of trauma, which is central to our study, but we recognize that some readers may perceive this as too generalized. We will revise the introduction to make it more concise and focused, ensuring that the primary aim of our study is clearer from the outset, while retaining the necessary background to contextualize the research. We aim to strike a balance between presenting the wider issue and focusing on the specific research we report in this paper. We appreciate your constructive feedback, which will help us improve the clarity of the introduction. Finally, we also include this sentence to clarify to the readers: *Nevertheless, it is important to note, that while early-life adversity can have significant long-term effects on the welfare of macaques, it would be inappropriate to generalise these findings to all laboratory settings. In recent years, there has been increasing recognition of the need for improved welfare standards for primates in research, with more stringent regulations and ethical guidelines now in place to ensure better living conditions and care. These advances in animal welfare practices aim to mitigate the negative impacts of early experiences, making it essential to avoid an overly broad characterisation of all laboratory environments as inherently detrimental.*

 Beyond that, probably I would suggest to simplify the tables and present the numbers of the statistics in a supplementary part. Furthermore, do not refer to Table 1 for explanations that can be directly inserted in the text.

 Response: Thank you for your suggestion. We have already moved a significant portion of the tables, including statistical details, to the supplementary materials section to streamline the main text. Regarding the explanations, we prefer to refer to Table 1 for consistency and clarity, as it allows us to maintain a concise narrative in the main text while still providing detailed information in an accessible format for readers.

 I congratulate with the authors for this impressive and important research.

 Response: Thank you very much for your kind words and congratulations. We truly appreciate your positive feedback, and we are glad that you found the research valuable and impactful.

# Reviewer report: Reviewer response for version 1

**DOI:** 10.5256/f1000research.157991.r294421

**Published:** 2024-08-26

**Authors:** Lief Erikson Gamalo

**Affiliations:** 1 University of the Philippines Mindanao, Davao City, Philippines

This paper provides a valuable contribution to the field and advances our understanding on impacts of trades on macaques' socio-emotional and behavioral skills. The research uses thorough methodology and analysis. Given its potential impacts to the field of conservation and animal welfare, this important piece of work is recommended to be indexed. 

 Here are my comments and suggestions;

 1. Authors should add authority in the first mention for each scientific names.

 2. **Page 3:** " *...marmosets, Rhesus and long-tailed*

*macaques...*" should be ". *..marmosets, Rhesus, and long-tailed macaques*"

 3. **Page 4:** " *LCTW, a former zoo converted into an animal rescue and rehabilitation centre."* should be " *LCTW is a former zoo that has been converted into an animal rescue and rehabilitation centre*."

 4. **Page 5: **" *We thus focused on the study of 53 focal subjects within a broader sample of 88 macaques. Focal animals were ranged in ages between 1 and 18 years old.......ranging from 10 to 13 years old *". This paragraph is a little bit confusing. Maybe the authors can find a better way of presenting this?

 5. **Page 9:** " *The SRS [short version] scale comprise....*". Delete " *[short version]*"

 6. **Pages 10-16: **Starting with the subheading social responsiveness in Result section. To me, this portion distracted the main focus of the paper. Thus, I would suggest to make a separate section about the statistical process and results of the reliability of the questionnaire labeling it as questionnaire reliability or related terms. Make it concise.

 7. **Figures 1-12:** I recommend that the authors should place the meaning of those codes in the figure description, instead of referring the readers to table 1.

Is the work clearly and accurately presented and does it cite the current literature?

Yes

If applicable, is the statistical analysis and its interpretation appropriate?

I cannot comment. A qualified statistician is required.

Are all the source data underlying the results available to ensure full reproducibility?

Yes

Is the study design appropriate and is the work technically sound?

Yes

Are the conclusions drawn adequately supported by the results?

Yes

Are sufficient details of methods and analysis provided to allow replication by others?

Yes

Reviewer Expertise:

Wildlife and conservation

I confirm that I have read this submission and believe that I have an appropriate level of expertise to confirm that it is of an acceptable scientific standard, however I have significant reservations, as outlined above.

# Response

**Published:** 2024-11-11

**Authors:** Miquel Llorente

**Affiliations:** Psychology, University of Girona, Girona, Catalonia, Spain

This paper provides a valuable contribution to the field and advances our understanding on impacts of trades on macaques' socio-emotional and behavioral skills. The research uses thorough methodology and analysis. Given its potential impacts to the field of conservation and animal welfare, this important piece of work is recommended to be indexed. 

**Response: **Thank you very much for your kind and encouraging feedback. We are delighted to hear that you find the study valuable and recognise its contribution to the fields of conservation and animal welfare **.**

 Here are my comments and suggestions;

 1. Authors should add authority in the first mention for each scientific names.

**Response: **Thank you very much for your thoughtful suggestion regarding the inclusion of the authority for each scientific name. We appreciate your attention to detail. However, we have chosen not to include these, as it is uncommon in primatology literature to do so. We hope this approach aligns with standard practice and maintains clarity for our readers

 2. **Page 3:** " *...marmosets, Rhesus and long-tailed*

*macaques...*" should be ". *..marmosets, Rhesus, and long-tailed macaques*"

**Response: **Thank you, we included it.

 3. **Page 4:** " *LCTW, a former zoo converted into an animal rescue and rehabilitation centre."* should be " *LCTW is a former zoo that has been converted into an animal rescue and rehabilitation centre*."

**Response: **We modified the sentence as: “LCTW is a former zoo that has been transformed into an animal rescue and rehabilitation centre, currently housing over one hundred individuals from various macaque species, which were victims of poaching”.

 4. **Page 5: **" *We thus focused on the study of 53 focal subjects within a broader sample of 88 macaques. Focal animals were ranged in ages between 1 and 18 years old.......ranging from 10 to 13 years old *". This paragraph is a little bit confusing. Maybe the authors can find a better way of presenting this?

**Response: **We modified as: “In this study we  investigated 53 focal subjects selected from a larger sample of 88 macaques. These focal animals, aged between 1 and 18 years old (mean age ± SD = 8 ± 5 years),  were distributed across eleven groups/enclosures (Suppl. Table 1 in the extended data for biographic information).”

 5. **Page 9:** " *The SRS [short version] scale comprise....*". Delete " *[short version]*"

**Response: **Thank you for your observation. If possible, we would prefer to maintain '[short version]' to clearly differentiate it from the long version of the SRS, as this distinction is relevant for our methodology.

 6. **Pages 10-16: **Starting with the subheading social responsiveness in Result section. To me, this portion distracted the main focus of the paper. Thus, I would suggest to make a separate section about the statistical process and results of the reliability of the questionnaire labeling it as questionnaire reliability or related terms. Make it concise.

**Response: **Thank you for your valuable suggestion. We understand your concern regarding the potential distraction from the main focus of the paper. However, we prefer to maintain these sections and explanations, as we consider them necessary for understanding the subsequent statistical processes. To simplify the main text, we have moved a significant portion of the ICC tables, factor analysis, and item scores to the supplementary materials section

 7. **Figures 1-12:** I recommend that the authors should place the meaning of those codes in the figure description, instead of referring the readers to table 1.

**Response: **Thank you for your suggestion. While we understand your point, we prefer not to make this change as the meaning of each code may be quite lengthy and would not fit within the design of the figures. To maintain consistency throughout the manuscript, we have opted to continue using the codes in all figures and refer readers to Table 1 for their full explanation. Additionally, the other three reviewers have expressed no concerns about this design choice

# Reviewer report: Reviewer response for version 1

**DOI:** 10.5256/f1000research.157991.r294419

**Published:** 2024-06-28

**Authors:** Luciana Oklander

**Affiliations:** 1 Grupo de Investigación en Genética Aplicada (GIGA, IBS, UNAM-CONICET, Argentina)., CONICET Nordeste, Posadas, Misiones, Argentina

It is an exceptional work; it contributes a lot to science and knowledge and above all it is novel in terms of approach and very necessary. All of us who work or are in contact with rescue centers know that animals that have been pets are very difficult (or impossible) to rehabilitate in order to be parte of a serious reintroduction or population reinforcement project, but this knowledge was not supported to my knowledge, until now by scientific studies. It was also a saying that the younger you rescue them, the more possibilities to rehab, but this study scientifically demonstrates it.

 However, it seems to me that the manuscript would need some adjustments in the abstract so it is understood precisely how the work was carried out. On the results a strong reduction of the text and of the tables presented is needed, making them more understandable for any reader in the form of graphs or histograms.

**Abstract**

 Add that 53 focal subjects were studied within the resident macaques at Lao Conservation Trust for Wildlife center (LCTW). LCTW, a former zoo converted into an animal rescue and rehabilitation centre.

 When you mention social exposure, say that you are referring to individuals of the same species (if that is the case).

*“The lack of juvenile social contact*

*predicts reduced social behaviours and an inclination towards social*

*avoidance in adulthood. Macaques raised by humans tend to exhibit*

*more abnormal behaviours in social contexts, compromising their*

*welfare. Deprivation of social exposure in infancy negatively impacts*

*psychological stimulation and overall welfare. The duration of time*

*spent in illegal trade correlates with heightened anxiety levels.*

*Personality traits such as ‘Calmness’ and ‘Unfriendliness’ are*

*influenced by rearing conditions, with macaques deprived of social*

*interaction during their early years showing higher levels of*

*introversion. In conclusion, the absence of social exposure during*

*early life and hand-rearing due to illegal trade significantly shape”*

 All of this could be summarized in a paragraph that makes it clear that the more human contact and the less with their own kind, anxiety levels intensify, etc., as is very well written in the conclusions.

**Introduction**

*adverse childhood experiences (ACE) early adverse rearing experiences (EARE)*, what is the difference between these?

*socio-emotional and behavioural (SEB)*. please mention this without the acronyms

 “ *and four secondary goals*” wich ones?

 “ *deprivation of social interactions*”, always clarify with individuals of the same species

**Methods**

 “ *Focal animals were ranged in ages between 1 and 18 years old*.” Add here what the ages of infant/adolescent and adult are in each of these species.

*“Focal animals were ranged in ages between 1 and 18 years old (mean age* *_* *SD = 8* *_* *5 years) and were distributed across eleven groups/enclosures(Suppl. Table 1 in the extended data): P1 (651,56 m* *2* *) with 7 stump-tailed macaques (* *Macaca arctoides)* *, consisting of 3 females and 4 males, with estimated ages ranging from 8 to 21 years old; P2 (653,76m* *2* *) with 8 stump-tailed macaques,including 4 females and 4 males, ranging in age from 1 to 15 years old; P4 (949,77 m* *2* *) with 3 Rhesus (* *Macaca mulatta* *),1 Assamese (* *Macaca assamensis* *) and 11 Northern pig-tailed macaques (* *Macaca leonina* *) [15 in total], of which7 females and 8 males with estimated ages ranging from 1 to 11 years old; P5 (1236,63 m* *2* *) with 8 Rhesus, 9 Assamese,1 pig-tailed and 1 long-tailed macaque (* *Macaca fascicularis* *) [19 in total], of which 9 females and 10 males withestimated ages ranging from 11 months to 11 years old; P6 (874,49 m* *2* *) with 3 Rhesus and 5 Assamese macaques [8 intotal], comprising 4 females and 4 males with estimated ages ranging from 5 to 10 years old; P7 (705,08 m* *2* *) with 7 pigtailed macaques, consisting of 4 females and 3 males with estimated ages ranging from 3 to 8 years old; P8 (340,04 m* *2* *)*

*with 5 stump-tailed macaques of which 3 females and 2 males, with estimated ages ranging s from 10 months to 17 years*

*old; P9 (233,03m* *2* *) with 5 stump-tailed macaques, consisting of which 3 females and 2 males with estimated ages ranging*

*from 5 to 8 years old; P10 (51,72 m* *2* *) with 2 Assamese, 3 long-tailed and 2 pig-tailed macaques [7 in total], comprising*

*3 females and 5 males with estimated ages ranging from 2 to 12 years old; BP1 (50-100m* *2* *) with 3 pig-tailed macaques of*

*which 1 female and two males with estimated ages ranging from 7 to 10 years old; and BP3 (50-100 m* *2* *) with 1 pig-tailed*

*and 3 Assamese macaques [4 in total], consisting of 1 female and 3 males with estimated ages ranging from 10 to* *13 years old.”*

 All this is very difficult to follow, I suggest making a table with species, with number of individuals detailed in columns sex and age and what groups/enclosures it was in, but as Table 1 in the manuscript, not as supplementary.

 in “Table 1”: *peers* means same species, or could be another one? Please clarify in each case

 “ *Three human raters were*…” Human sounds strange here I would say caregiver or something like that

**Results**

 From tables 3 to 14 I believe that the results can be shown in column graphs or cakes with the significance, but the detailed numbers from these tables should be presented as supplementary.

 For the figures presented, please put what it means directly in the footer and do not redirect to Table 1.

Is the work clearly and accurately presented and does it cite the current literature?

Yes

If applicable, is the statistical analysis and its interpretation appropriate?

Yes

Are all the source data underlying the results available to ensure full reproducibility?

Yes

Is the study design appropriate and is the work technically sound?

Yes

Are the conclusions drawn adequately supported by the results?

Yes

Are sufficient details of methods and analysis provided to allow replication by others?

Yes

Reviewer Expertise:

Behavior, genetics, ecology and conservation of primates

I confirm that I have read this submission and believe that I have an appropriate level of expertise to confirm that it is of an acceptable scientific standard, however I have significant reservations, as outlined above.

# Response

**Published:** 2024-11-11

**Authors:** Miquel Llorente

**Affiliations:** Psychology, University of Girona, Girona, Catalonia, Spain

**Thank you for your positive feedback. We are pleased that you have recognised the significance and novelty of our work. As you highlight, our research scientifically confirms the difficulty of rehabilitating animals rescued from human care for release. We hope our findings will contribute to improving rescue and rehabilitation practices in the field.**

 However, it seems to me that the manuscript would need some adjustments in the abstract so it is understood precisely how the work was carried out. On the results a strong reduction of the text and of the tables presented is needed, making them more understandable for any reader in the form of graphs or histograms.

**Abstract**

 Add that 53 focal subjects were studied within the resident macaques at Lao Conservation Trust for Wildlife center (LCTW). LCTW, a former zoo converted into an animal rescue and rehabilitation centre.

 When you mention social exposure, say that you are referring to individuals of the same species (if that is the case).

**We included the suggestion and details in the abstract.**

*“The lack of juvenile social contact*

*predicts reduced social behaviours and an inclination towards social*

*avoidance in adulthood. Macaques raised by humans tend to exhibit*

*more abnormal behaviours in social contexts, compromising their*

*welfare. Deprivation of social exposure in infancy negatively impacts*

*psychological stimulation and overall welfare. The duration of time*

*spent in illegal trade correlates with heightened anxiety levels.*

*Personality traits such as ‘Calmness’ and ‘Unfriendliness’ are*

*influenced by rearing conditions, with macaques deprived of social*

*interaction during their early years showing higher levels of*

*introversion. In conclusion, the absence of social exposure during*

*early life and hand-rearing due to illegal trade significantly shape”*

 All of this could be summarized in a paragraph that makes it clear that the more human contact and the less with their own kind, anxiety levels intensify, etc., as is very well written in the conclusions.

**Thank you for your valuable feedback. I have revised the abstract to incorporate your suggestion, summarising the findings in a final paragraph that highlights how increased human contact and reduced exposure to conspecifics correlate with intensified anxiety, abnormal social behaviours, and impacted personality traits. I believe this adjustment clarifies the main conclusions and enhances the overall coherence of the abstract**

 Introduction

*adverse childhood experiences (ACE) early adverse rearing experiences (EARE)*, what is the difference between these?

**Thank you for your question. 'Adverse Childhood Experiences' (ACE) is a term primarily used in human-focused literature to describe traumatic or negative experiences during childhood. In contrast, 'Early Adverse Rearing Experiences' (EARE) is commonly used in animal research to refer to similar early-life adversities in non-human subjects, such as macaques. This distinction helps to contextualise findings within the appropriate body of literature for each field.**

*socio-emotional and behavioural (SEB)*. please mention this without the acronyms

**Thank you for the observation. We have removed the acronym, and 'socio-emotional and behavioural' is now written in full throughout the text**

 “ *and four secondary goals*” wich ones?

**Thank you for your insightful comment. We have revised the objectives section to eliminate the specific mention of primary and secondary goals, presenting the research aims in a more integrated manner. I believe this change enhances the clarity and flow of the text.**

 “ *deprivation of social interactions*”, always clarify with individuals of the same species

**Thank you for your suggestion. We have revised the text to clarify that 'deprivation of social interactions' refers specifically to interactions with individuals of the same species. We implemented this change  consistently throughout the manuscript.**

 Methods

 “ *Focal animals were ranged in ages between 1 and 18 years old*.” Add here what the ages of infant/adolescent and adult are in each of these species.

*“Focal animals were ranged in ages between 1 and 18 years old (mean age _ SD = 8 _ 5 years) and were distributed across eleven groups/enclosures(Suppl. Table 1 in the extended data): P1 (651,56 m2) with 7 stump-tailed macaques (Macaca arctoides), consisting of 3 females and 4 males, with estimated ages ranging from 8 to 21 years old; P2 (653,76m2) with 8 stump-tailed macaques,including 4 females and 4 males, ranging in age from 1 to 15 years old; P4 (949,77 m2) with 3 Rhesus (Macaca mulatta),1 Assamese (Macaca assamensis) and 11 Northern pig-tailed macaques (Macaca leonina) [15 in total], of which7 females and 8 males with estimated ages ranging from 1 to 11 years old; P5 (1236,63 m2) with 8 Rhesus, 9 Assamese,1 pig-tailed and 1 long-tailed macaque (Macaca fascicularis) [19 in total], of which 9 females and 10 males withestimated ages ranging from 11 months to 11 years old; P6 (874,49 m2) with 3 Rhesus and 5 Assamese macaques [8 intotal], comprising 4 females and 4 males with estimated ages ranging from 5 to 10 years old; P7 (705,08 m2) with 7 pigtailed macaques, consisting of 4 females and 3 males with estimated ages ranging from 3 to 8 years old; P8 (340,04 m2)*

*with 5 stump-tailed macaques of which 3 females and 2 males, with estimated ages ranging s from 10 months to 17 years*

*old; P9 (233,03m2) with 5 stump-tailed macaques, consisting of which 3 females and 2 males with estimated ages ranging*

*from 5 to 8 years old; P10 (51,72 m2) with 2 Assamese, 3 long-tailed and 2 pig-tailed macaques [7 in total], comprising*

*3 females and 5 males with estimated ages ranging from 2 to 12 years old; BP1 (50-100m2) with 3 pig-tailed macaques of*

*which 1 female and two males with estimated ages ranging from 7 to 10 years old; and BP3 (50-100 m2) with 1 pig-tailed*

*and 3 Assamese macaques [4 in total], consisting of 1 female and 3 males with estimated ages ranging from 10 to 13 years old.”*

 All this is very difficult to follow, I suggest making a table with species, with number of individuals detailed in columns sex and age and what groups/enclosures it was in, but as Table 1 in the manuscript, not as supplementary.

**In response to your suggestion, I have created a table that clearly presents the details of the focal animals, including species, group/enclosure, number of individuals, sex distribution, and age range. This table will be included as Table 1 in the manuscript for clarity and ease of reference. Thank you for your helpful feedback!**

 in “Table 1”: *peers* means same species, or could be another one? Please clarify in each case

**Yes, it means same species. We replace “peers” by “conspecifics” in the table.**

 “ *Three human raters were*…” Human sounds strange here I would say caregiver or something like that

**Thank you for the suggestion. We have removed "human" and kept "three raters" only, as these raters can be either veterinarians or researchers.**

**Results**

 From tables 3 to 14 I believe that the results can be shown in column graphs or cakes with the significance, but the detailed numbers from these tables should be presented as supplementary.

**We move tables 3 to 14 to supplementary materials as suggested by the reviewer, now renamed as tables 12 to 24.**
